# Petiole-Lamina Transition Zone: A Functionally Crucial but Often Overlooked Leaf Trait

**DOI:** 10.3390/plants10040774

**Published:** 2021-04-15

**Authors:** Max Langer, Thomas Speck, Olga Speck

**Affiliations:** 1 Plant Biomechanics Group @ Botanic Garden Freiburg, University of Freiburg, D-79104 Freiburg, Germany; thomas.speck@biologie.uni-freiburg.de (T.S.); olga.speck@biologie.uni-freiburg.de (O.S.); 2 Cluster of Excellence livMatS @ FIT—Freiburg Center for Interactive Materials and Bioinspired Technologies, University of Freiburg, D-79110 Freiburg, Germany

**Keywords:** transition zone, foliage leaf, petiole, lamina, anatomy, morphology, *Pilea peperomioides*, *Hemigraphis alternata*, *Hosta x tardiana* ‘El Niño’, *Caladium bicolor*

## Abstract

Although both the petiole and lamina of foliage leaves have been thoroughly studied, the transition zone between them has often been overlooked. We aimed to identify objectively measurable morphological and anatomical criteria for a generally valid definition of the petiole–lamina transition zone by comparing foliage leaves with various body plans (monocotyledons vs. dicotyledons) and spatial arrangements of petiole and lamina (two-dimensional vs. three-dimensional configurations). Cross-sectional geometry and tissue arrangement of petioles and transition zones were investigated via serial thin-sections and µCT. The changes in the cross-sectional geometries from the petiole to the transition zone and the course of the vascular bundles in the transition zone apparently depend on the spatial arrangement, while the arrangement of the vascular bundles in the petioles depends on the body plan. We found an exponential acropetal increase in the cross-sectional area and axial and polar second moments of area to be the defining characteristic of all transition zones studied, regardless of body plan or spatial arrangement. In conclusion, a variety of terms is used in the literature for describing the region between petiole and lamina. We prefer the term “petiole–lamina transition zone” to underline its three-dimensional nature and the integration of multiple gradients of geometry, shape, and size.

## 1. Introduction

### 1.1. Damage-Resistant Transition

Leaves, which are the main site of photosynthesis, can differ considerably in their geometry, shape, size, and venation. In most leaves, the planar leaf blade (=lamina), which is often connected to the stem by a rod-shaped leaf stalk (=petiole), captures the light needed to produce energy-rich organic molecules. On this basis, the loss of leaf blades, e.g., as caused by drought stress, frost, diseases, or mechanical damage, poses an existential threat to the plant. Thus, one can assume that a high selective pressure exists on the development of a damage-resistant transition between the lamina and petiole during evolution.

### 1.2. A Bunch of Terms

A literature search shows that the transition between petiole and lamina does not have a common name, which is possibly one of the reasons that it is not mentioned in modern textbooks. Table 1 shows a compilation of selected publications that have addressed this topic from various scientific perspectives. Some of the terms used express the particular aspect of a specific spatial arrangement: “point” is to be understood as one-dimensional, “area” as two-dimensional, and “zone” as three-dimensional. Other terms describe the quality of the connection between the petiole and lamina: “transition” expresses the occurrence of gradients, “union” stands for a fusion created during ontogenesis, “attachment” includes a hierarchical aspect, “junction” is the place at which the petiole and lamina join, “juncture” represents the seam at which they are joined, “entrance” is the direction in which one passes over into the other, “border” is the separating line, and “intersection” is the place at which the petiole and lamina cross.

Probably, the chosen terms are related to various scientific perspectives, such as systematics, morphology, anatomy, biomechanics, and biomimetics and to whether the whole leaf or selected aspects such as the venation have been investigated. Sewell [1] described the connection of the petiole and lamina from an ontogenetic viewpoint, in particular from observations after germination of various *Salvia* species. Niinemets and Fleck [6] pursued a biomechanical point of view and considered both the petiole and midrib of the lamina as individual beam-like structures that are connected to each other and that mechanically influence one another. Poulsen and Nordal [7] approached the topic from a systematic and morphometric perspective. They classified various *Chlorophytum* species according to their petiole and lamina characteristics, thus explicitly distinguishing between stalk and blade. Jones and Kang [9] investigated the ontogenetic/developmental pattern formation of leaf veins anatomically including the transition from the petiole to lamina. In the framework of a biomimetic project, Langer et al. [12] investigated the petiole–lamina transition zone of foliage leaves as models for technical solutions between rod-shaped and planar elements in architecture. They have shown that the cross-sectional shapes of the peltate leaves of *Caladium bicolor* gradually change from an almost circular petiole to a triangular transition zone and, finally, to a three-lobed star-shaped planar lamina. Sacher et al. [13] examined the transition zones of the peltate leaves of *Colocasia fallax* and *Tropaeolum majus*, showing how the transition between lamina and petiole handles mechanical stress and the important role of the vascular bundles for load dissipation.

### 1.3. Materials Systems with Multiple Gradients

In general, plants can be regarded as fiber-reinforced materials systems, such that stiff and rigid tissues such as vascular bundles and fibers are embedded in a more flexible matrix of parenchyma [19,20]. By analogy to functionally graded materials in technology, plants maintain resistance to damage by spatial gradients rather than by abrupt changes. Spatial gradients can effectively enhance the functionality and biomechanical performance of biological materials systems by alleviating stress concentrations, allowing the formation of new functions and enabling adaptations to partially conflicting requirements [21,22]. An impressive example for the development of such a compromise are foliage leaves, which show an interplay of flexural rigidity in order to bear the weight of the leaf and torsional flexibility (respectively low torsional rigidity) in order to enable streamlining upon wind loads. Flexural rigidity (EI) is composed of the bending elastic modulus (E) and the axial second moment of area (I), whereas torsional rigidity (GJ) is composed of the torsional modulus (G) and the polar second moment of area (J). Vogel [23] has characterized this trade-off by defining the dimensionless twist-to-bend ratio (EI/GJ).

The integration of multiple gradients can take place on various hierarchical levels in the “bottom-up” manner from molecules to cells and tissues and further to plant organs and entire plants [22]. According to Liu et al. [21], the gradients in biological materials systems are fundamentally associated with changes in (i) structural characteristics including the arrangement, distribution, dimensions, and orientations of building units, (ii) chemical compositions between similar components, and (iii) gradient interfaces between dissimilar components.

Various gradients are present in foliage leaves. A gradient in arrangement is reflected by the architecture in the zone between the petiole and lamina. According to their spatial arrangement, we distinguish between the 3D-configuration (angle approx. 90°) as in peltate leaves and between the 2D-configuration (angle approx. 0°) as in leaves with a petiole connected to the basal lamina margin.

In the context of the flexural and torsional loading of the petioles, the gradient in dimension is the continuous increase or decrease of the cross-sectional area (A), axial second moment of area (I) and polar second moment of area (J). The ratio of I/J provides information about the influence of the different cross-sectional geometries and their shapes to the twist-to-bend ratio [23,24,25]. Generally, the I/J ratio is 0.25 for elliptical cross-sections if the minor axis is half the major axis (minor axis aligned in the vertical direction), 0.2–0.5 for U-profiled cross-sections, 0.5 for circular cross-sections, 0.81 for squared cross-sections, 0.83 for isosceles triangles, and 1.25 for elliptical cross-sections if the major axis is twice the minor axis (major axis aligned in the vertical direction). Consequently, high values of EI/GJ can usually be found if the bending elastic modulus (E) is much higher than the shear modulus (G) [26].

The gradient of distribution is mirrored by the increasing or decreasing density of vascular bundles and fibers in the periphery or the center of the cross-section, respectively. The distribution of strengthening tissues has a pronounced influence on the axial and polar second moments of area of these tissues. In principle, the cross-sectional distribution of the vascular bundles is fixed by the body plan of plants, which is a set of morphological features common to many members of a phylum (also termed “bauplan” [27]). In general, monocotyledons have an atactostele and dicotyledons have an eustele. Deviations from the general body plan are possible with respect to the specific functions of the leaves, such as water and food storage, attachment, or defense.

The gradient in orientation is based on anisotropic structural units with properties that depend strongly on orientation [21,22]. A gradual reorientation of the fibers and vascular bundles in the transition between petiole and lamina depends on the body plan and, thus, on parallel, reticulate or radiate venation and always contributes markedly to the damage-resilient connection of both leaf parts. In addition, this gradual reorientation is also dependent on the spatial configuration of the petiole and lamina, as it differs between the 2D- and 3D-configurations of leaves.

For plants, the phenolic polymer lignin plays a crucial role for spatial gradients attributable to the variations in the chemical composition at the interface between dissimilar components, a point at which contact failure commonly occurs [28]. In most cases, strengthening tissues are directly surrounded by a lignin gradient in the cell walls of the surrounding parenchymatous cells [29,30]. This lignification gradient prevents abrupt changes at the interface between non-lignified parenchyma with an elastic modulus of 5 to 100 MPa and lignified sclerenchyma fibers with an elastic modulus of 24 to 45 GPa [31].

### 1.4. Motivation

Although both the petiole and lamina of foliage leaves have been well studied in terms of their functional morphology and biomechanics, a detailed analysis of the transition zone between them has barely been addressed. The scientific question of this study has been to identify objectively measurable morphological and anatomical criteria for a generally valid definition and characterization of the petiole–lamina transition zone of foliage leaves by means of a comparative study of four model plants with various body plans (mono- and dicotyledons) and leaf architectures (peltate leaves and leaves with a marginal petiole position). Four main aspects for distinguishing between the petiole, transition zone and lamina have been assessed: (i) the changes of cross-sectional geometry, (ii) the determination of shape (e.g., aspect ratio, tapering mode, ratio of axial and polar second moment of area), (iii) the quantification of size (e.g., linear or exponential increase of cross-sectional area), and (iv) the three-dimensional arrangement and course of the vascular bundles (e.g., number and contribution to cross-sectional area of vascular bundles).

## 2. Results

**Figure 1:**
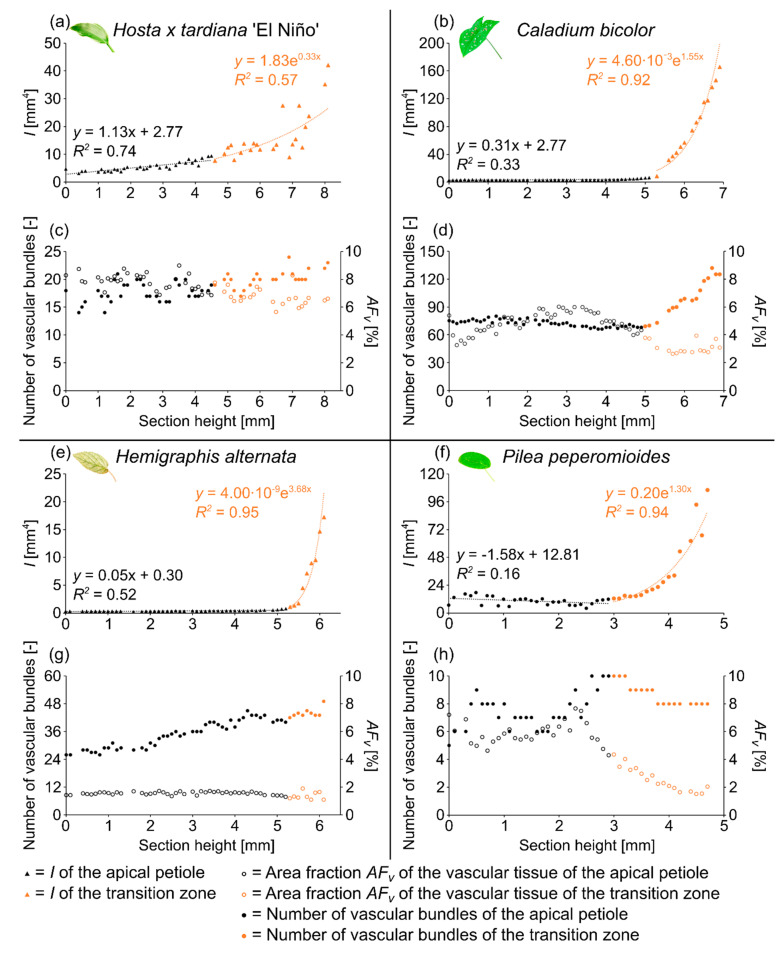
Axial second moment of area *I* (**a**,**b**,**e**,**f**), number of vascular bundles, and area fraction of the vascular tissue *AF_v_* (**c**,**d**,**g**,**h**) of the apical petiole and the transition zone of one leaf of *Hosta x tardiana* ‘El Niño’ (**a**,**c**), *Caladium bicolor* (**b**,**d**), *Hemigraphis alternata* (**e**,**g**), and *Pilea peperomioides* (**f**,**h**) plotted against the section height (0 = basal end of the samples).

The four species studied show similarities and dissimilarities with respect to their general body plan as monocotyledons and dicotyledons and their spatial arrangement of the petiole and lamina. According to Niklas [32], geometry, shape, and size are not synonymous. Size, for example, is a substantial variable that can be expressed in units of a physical quantity, whereas shape is a natural variable that has a magnitude but lacks a unit. In the present study, we distinguish among various cross-sectional geometries (e.g., circle, ellipse, or triangle) and describe “shape” by calculating the aspect ratio AR and the tapering mode α (Table 2). Moreover, we measure “size” in terms of the cross-sectional area A, the axial second moment of area I, and the polar second moment of area J. In the following, we present these quantitative results in Table 3 and some of these results exemplarily for one sample of each species studied in Figure 1.

**Figure 2:**
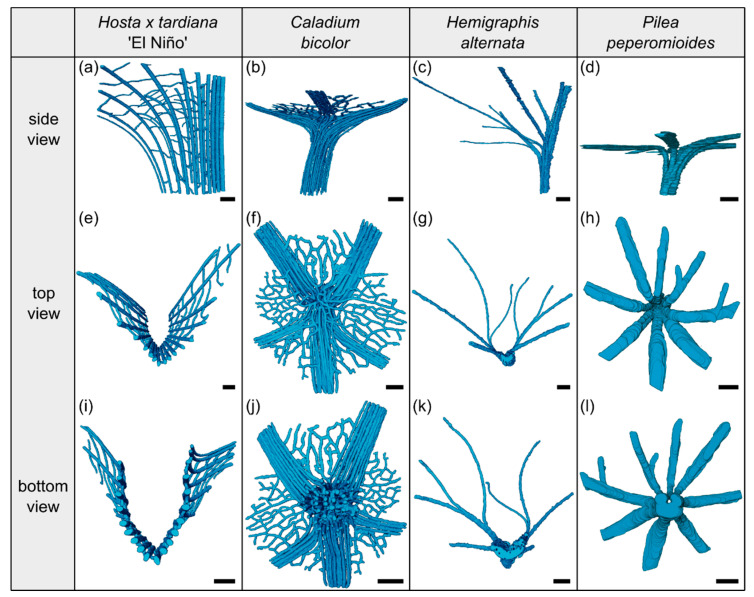
Segmented vascular tissue from µCT data of the transition zones of *Hosta x tardiana* ‘El Niño’ (**a**,**e**,**i**), *Caladium bicolor* (**b**,**f**,**j**), *Hemigraphis alternata* (**c**,**g**,**k**), and *Pilea peperomioides* (**d**,**h**,**l**). A side, top, and bottom view of the vascular tissue is shown for each species. In side view, the specimens are oriented with the abaxial side to the right and the adaxial side to the left. Scale bars for all images equal 1 mm.

In addition, we display in Figure 2 the µCT results of the three-dimensional arrangement of the vascular bundles in the petiole–lamina transition zone of all four investigated species. Videos of the µCT scans can be found in the Supplementary Materials Videos S1–S4.

**Figure 3:**
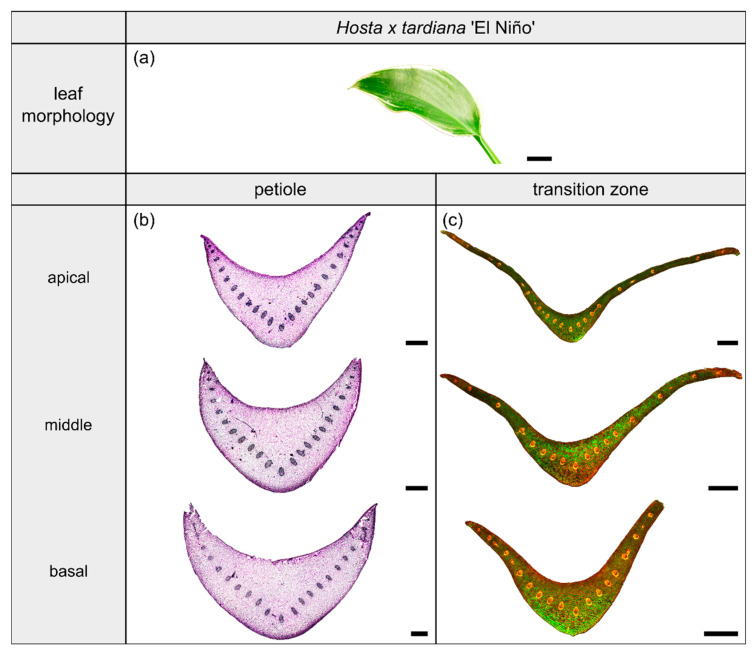
Morphology and anatomy of the foliage leaf of *Hosta x tardiana* ‘El Niño’ (**a**). The thin-sections of the transition zone are stained with acridine orange (**c**), while the thin-sections of the petiole are stained with toluidine blue (**b**). The scale bar of the leaf morphology equals 2 cm and those of the anatomical sections equal 1 mm.

**Figure 4:**
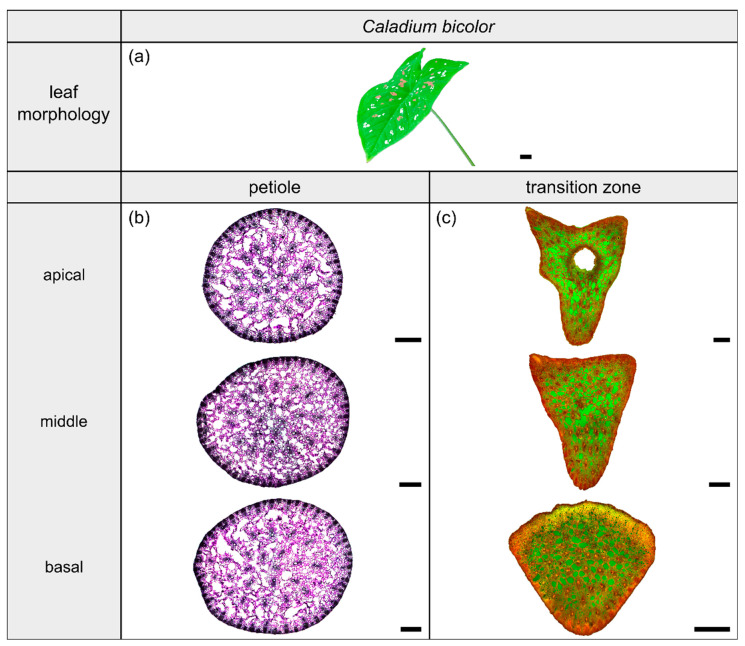
Morphology and anatomy of the foliage leaf of *Caladium bicolor* (**a**). The thin-sections of the transition zone are stained with acridine orange (**c**). The thin-sections of the petiole are stained with toluidine blue (**b**). The scale bar of the leaf morphology equals 2 cm and those of the anatomical sections equal 1 mm.

**Figure 5:**
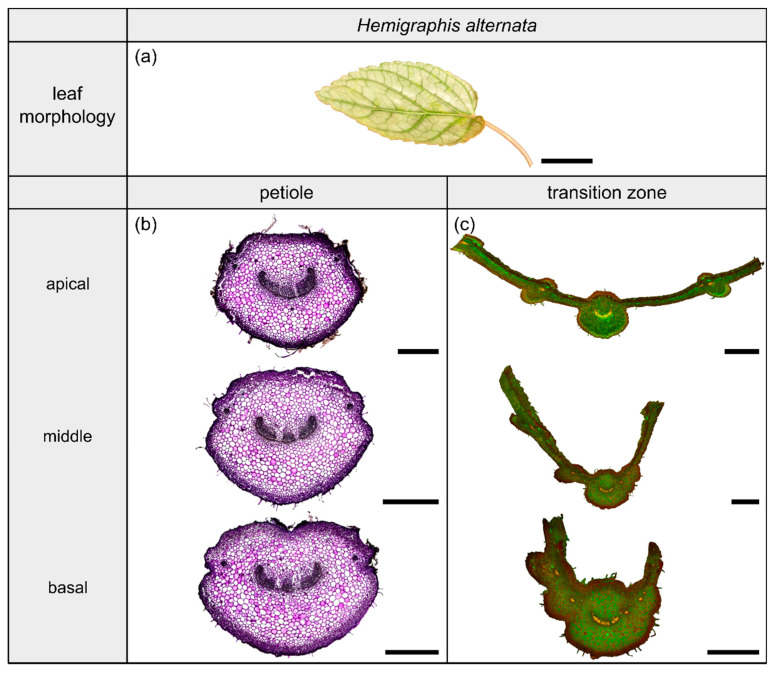
Morphology and anatomy of the foliage leaf of *Hemigraphis alternata* (**a**). The thin-sections of the transition zone are stained with acridine orange (**c**), whereas the thin-sections of the petiole are stained with toluidine blue (**b**). The scale bar of the leaf morphology equals 2 cm and those of the anatomical sections equal 1 mm.

**Figure 6:**
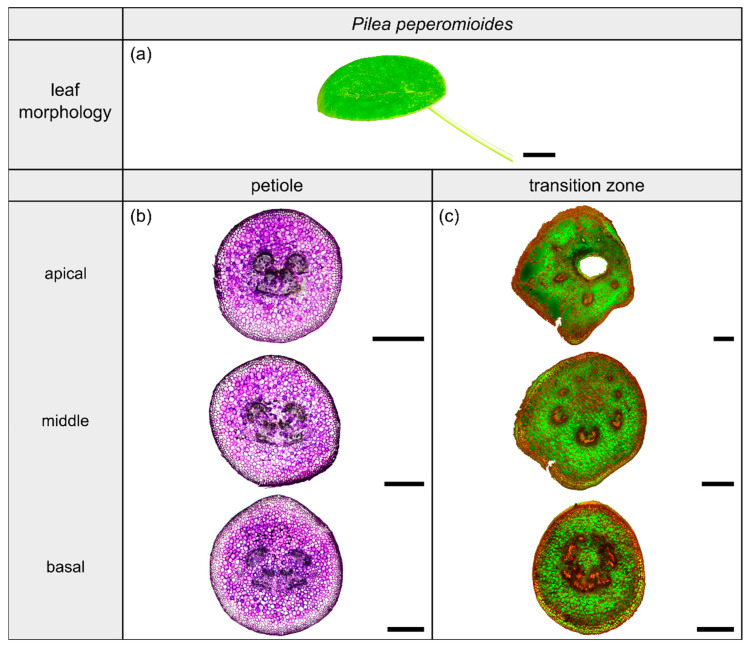
Morphology and anatomy of the foliage leaf of *Pilea peperomioides* (**a**). The thin-sections of the transition zone are stained with acridine orange (**c**) and those of the petiole are stained with toluidine blue (**b**). The scale bar of the leaf morphology equals 2 cm and those of the anatomical sections equal 1 mm.

The light microscopic results of the cross-sectional tissue arrangements are shown in Figure 3, Figure 4, Figure 5 and Figure 6 in the corresponding sections for each species.

### 2.1. Hosta x tardiana ‘El Niño’

#### 2.1.1. General Description of the Leaf

*Hosta x tardiana* ‘El Niño’ Piet Warmerdam (patent PP14632) (hereafter *H. tardiana*) belongs to the monocotyledonous family Asparagaceae. This cultivar preferentially grows in shaded habitats on slightly moist soil, for example in humid forests or on shaded, damp steep slopes. Their leaves are not sessile and thus untypically have a leaf sheath that is functionally a petiole. The petiole is marginally attached to the lamina and therefore is categorized as having a 2D-configuration. The elliptical lamina has an attenuated lamina base and a parallel venation typical of monocotyledons (Figure 3a).

#### 2.1.2. Geometry, Shape and Size

The petiole has a U-profile in transverse section, with a prominent groove on the adaxial side (Figure 3b). The petiole tapers hyperbolically. The change of the U-profile of the petiole to a V-profile indicates the beginning of the transition zone. The I/J ratio of the transition zone is significantly higher than that of the petiole (W=120, p=0.023) (Table 3). The linear (to a good approximation) increase of A, I, and J in the apical part of the petiole changes into an exponential increase in the petiole–lamina transition zone (Figure 1a, Table 3). This increase is attributable to the thin lamina that emerges on the mediolateral tips of the V-profile of the sections (Figure 3c). The lamina portion grows markedly in size, whereas the U-profile of the midrib remains essentially unchanged.

#### 2.1.3. Vascular Tissue

The arrangement of the individual vascular bundles shows a V-profile in the petiole (Figure 3b, the bundles appear in dark violet) and a U-profile in the transition zone (Figure 3c, the bundles appear in orange). In the transition zone, the distance between the bundles increases acropetally (Figure 2a,e,i). This applies in particular to the mediolateral bundles, which enter into the lamina. This acropetal increase in the inter-bundle distance is a consequence of the apically increasing curvature of the mediolateral bundles (Figure 2a). In contrast, the bundles of the basal transition zone run straight and in parallel. Additionally, the µCT data clearly show crosslinks between the longitudinal vascular bundles, which are only partially visible in the thin-sections. Figure 1c shows the results of a detailed analysis in 100 µm steps of a single sample. The number of vascular bundles increases from 18 to 19 in the apical region of the petiole and from 19 to 23 in the transition zone. In parallel, the area fraction of the vascular tissue decreases with increasing number of vascular bundles in the transition zone.

### 2.2. Caladium bicolor

#### 2.2.1. General Description of the Leaf

The species *Caladium bicolor* Vent. (hereafter *C. bicolor*) belongs to the monocotyledonous family Araceae and possesses peltate leaves having a 3D-configuration. This species grows mainly in moist habitats for example along rivers or in swampy areas. The leaves have a heart-shaped lamina with the petiole being slightly offset adaxially. They exhibit a rotate venation, with the veins radiating from the entry point of the transition zone into the lamina (Figure 4a).

#### 2.2.2. Geometry, Shape and Size

The petiole of *C. bicolor* is a circular truncated cone that tapers nearly linearly (Table 3). The geometrical change from the circular petiole (Figure 4b) to the triangular transition zone (Figure 4c) is accompanied by a change from a linear increase of A, I, and J in the apical part of the petiole to an exponential increase in the petiole–lamina transition zone (Figure 1b, Table 3). The exponential increase is visible in the increasing size of the triangular transverse section, especially in the abaxial direction, because of lobe formation at the flank of the triangular shape. Due to the 3D nature of the lamina, a hole occurs in the middle of the thin-section in the apical transition zone (Figure 4c). The I/J ratio of the transition zone is not significantly higher than that of the petiole (W=110, p=0.085) (Table 3).

#### 2.2.3. Vascular Tissue

The vascular tissue is scattered and embedded in the non-lignified parenchyma of the petiole (Figure 4b, the vascular tissue appears in dark violet) and the transition zone (Figure 4c, the vascular tissue appears in orange). Individual vascular bundles run in parallel in the basal transition zone; several vascular bundles align in the middle of the transition zone and merge to three vascular strands in the apical transition zone, from where they enter the lamina (Figure 2b,j). In addition, individual bundles are distributed in the lamina in a net-like manner and thus simultaneously interconnect the vascular strands. This net-like vascular structure, in the apical transition zone, emerges from the innermost individual bundles coming from the basal transition zone (Figure 2f). The large vascular strand on the abaxial side in the apical transition zone consists of individual bundles from the outer abaxial side and the outer mediolateral sides of the basal transition zone (Figure 2b). Furthermore, in the apical transition zone, smaller abaxial clusters split off from the large strands. Moreover, the two large adaxial strands consist of outer and inner adaxial bundles originating from the basal transition zone. All these individual vascular strands enter the lamina with a specific curvature. This curvature is higher for the adaxial strands than for the abaxial ones, since the basal transition zone is slightly inclined abaxially. Figure 1d shows the results of a detailed analysis in 100 µm steps of a single sample. The number of vascular bundles decreases from 75 to 68 in the apical petiole and increases from 69 to 125 in the transition zone. In parallel, the area fraction of the vascular tissue decreases with the increasing number of vascular bundles in the transition zone.

### 2.3. Hemigraphis alternata

#### 2.3.1. General Description of the Leaf

The species *Hemigraphis alternata* (Burm.f.) T.Anderson (hereafter *H. alternata*) belongs to the dicotyledonous family Acanthaceae and its leaves have a 2D-configuration (Figure 5a). This species grows preferably in partial shaded to open habitats on moist soil. The leaves possess an ovate lamina with an oblique base and a pinnate venation. The cross-sectional shape of the petiole is preserved in the form of the midrib, whereas the lamina portions increase over the course of the transition zone. The leaves exhibit a reticulate venation.

#### 2.3.2. Geometry, Shape and Size

The petiole is elliptic in transverse section with a groove on the adaxial side that is especially pronounced in the basal part (Figure 5b) and tapers hyperbolically (Table 3). The petiole has an epidermis with a thick underlying hypodermis, consisting of three to four layers of small thick-walled collenchyma cells. The beginning of the petiole–lamina transition zone is characterized by the appearance of the lamina on one of the mediolateral sides of the petiole (Figure 5c). Typically for an oblique base, the lamina appears further apically on the other side. The transition from the petiole into the lamina is reflected in the change from a slightly linear decrease to an exponential increase of A, I, and J (Figure 2e, Table 3). The I/J ratio of the transition zone is not significantly smaller than that of the petiole (W=59, p=0.448) (Table 3).

#### 2.3.3. Vascular Tissue

The vascular bundles of *H. alternata* form a U-profiled strand in the center of the cross-section of the petiole (Figure 5b, the bundles appear in dark violet) and the transition zone (Figure 5c, the bundles appear in orange). In the transition zone, the area of the central vascular bundle decreases from basal to apical (Figure 2g,k). The thin-sections (Figure 5c) and the µCT data (Figure 2c) reveal that individual vascular bundles depart from this central vascular strand outwards in mediolateral direction to supply the lamina. Within the lamina, leaf ribs emerge around these individual bundles in the form of lamina thickenings. However, we have not seen any crosslinks between the bundles and/or the U-profiled central strand (Figure 2c,g,k). Figure 1e shows the results of a detailed analysis in 100 µm steps of a single sample. The number of vascular bundles increases from 26 to 40 in the apical petiole and from 42 to 49 in the transition zone. In parallel, the area fraction of the vascular tissue decreases with increasing number of vascular bundles in the transition zone.

### 2.4. Pilea peperomioides

#### 2.4.1. General Description of the Leaf

The dicotyledonous species *Pilea peperomioides* Diels (hereafter *P. peperomioides*) belongs to the Urticaceae family and possesses orbicular and peltate leaves, categorized as having a 3D-configuration. This species grows in shady and moist habitats on rocks, for example in forests or on ledges of cliffs. The petiole is slightly offset adaxially. The leaves have a rotate venation, with the veins radiating from the entry point of the transition zone into the lamina (Figure 6a).

#### 2.4.2. Geometry, Shape and Size

The petiole of *P. peperomioides* is a circular truncated cone that tapers hyperbolically (Table 3). The geometrical change from a circular (Figure 6b) to an elliptical cross-section indicates the beginning of the petiole–lamina transition zone (Figure 6c). This change from the petiole into the lamina is also reflected in the change from a linear increase to an exponential increase of A, I, and J (Figure 1f). The exponential increase of the cross-sectional area can also be seen in the transverse thin-sections, particularly in the lobes forming in the middle transition zone (Figure 6c). Because of the 3D nature of the lamina, a hole occurs in the middle of the thin-section in the apical transition zone (Figure 6c). The I/J ratio of the transition zone is not significantly smaller than that of the petiole (W=48, p=0.190) (Table 3).

#### 2.4.3. Vascular Tissue

In the petiole, the vascular bundles are present as six strands in the center and, in the acropetal direction, converge closer together and partly merge with each other (Figure 6b, the bundles appear in dark violet). At the entry to the basal transition zone, the thin-sections (Figure 6c, the bundles appear in orange) and the µCT data (Figure 2l) show that the vascular bundles form a single U-profiled strand that is thicker on the abaxial side than on the adaxial side and that splits acropetally into eight separate vascular strands that radiate in all directions into the lamina (Figure 2d,h). Since the petiole is slightly inclined to the abaxial side, the abaxial strands enter the lamina with less curvature than the strands on the adaxial side (Figure 2d). No crosslinks between the individual strands could be found. Figure 1h shows the results of a detailed analysis in 100 µm steps of a single sample. The number of vascular bundles increases from 5 to 10 in the apical petiole and decreases from 10 to 8 in the transition zone as some bundles merge together. In parallel, the area fraction of the vascular tissue decreases in the transition zone.

## 3. Discussion

The object of this study has been to find measurable morphological and anatomical criteria in order to objectively distinguish the transition zone from the petiole and lamina and to characterize this important but often overlooked part of foliage leaves. We have carried out a comparative study to determine key criteria, with respect to geometry, shape, and size that are generally independent of the respective body plans. Therefore, the study includes the body plans of monocotyledons and dicotyledons and the various spatial arrangements of petiole and lamina in terms of peltate leaves (3D-configuration) or leaves for which the petiole is marginally attached to the lamina (2D-configuration).

Since we have never observed any tearing of the lamina from the petiole within the transition zone, this zone seems to be quite damage-resistant. In this context, Liu et al. [21] point out that damage formation can be prevented by superimposing several local properties on each other, resulting in a structure with various gradients (arrangement, distribution, dimension, orientation, interface, composition). We therefore examined the transition zones in our comparative study with regard to some of these gradients.

### 3.1. Gradual Change of Geometry

All examined foliage leaves show a gradual change in cross-sectional geometry. The leaves with a 2D-configuration mostly retain the geometry of their petiole, with the latter continuously merging into the lamina as a midrib. Apically, the cross-sections are more and more characterized by the mediolaterally attached parts of the lamina. In contrast, in the leaves with a 3D-configuration, the geometry of the petiole is not preserved. The geometry changes from circular to triangular or elliptical and, finally, lobules of the lamina can be seen that emerge almost uniformly in all directions. Since the petiole and lamina meet at an angle of about 90°, the tissues of the petiole–lamina transition zone must also show a corresponding curvature.

In summary, the change of geometries from the petiole to the transition zone and midrib are more dependent on the spatial arrangement as a 2D- or 3D-configuration than on the body plans of the monocotyledons or dicotyledons.

### 3.2. Gradual Change of Shape

For all species analyzed in the present study, with the exception of *H. tardiana*, the ratio of axial and polar second moments of area (*I*/*J*) does not differ significantly on comparing the petiole and transition zone. This indicates that *I*/*J* is preserved in terms of a gradient of dimension. Both investigated species with a 2D-configuration possess an adaxial groove, whereas the species with a 3D-configuration do not. *H. alternata* has a tiny groove at the base of the petiole, whereas *H. tardiana* keeps a U-profile over the entire petiole including the transition zone. An individual deep groove can convert a circular profile with an *I*/*J* = 0.50 [33] into a U-profile. In the case of *H. tardiana*, the median *I*/*J* = 0.33 of the U-profiled petioles is significantly smaller than the median *I*/*J* = 0.45 of the V-profiled transition zones. In leaves with a 2D-configuration, the highest bending moments occur at the base of the petioles. An increase in flexural rigidity combined with adequate torsional rigidity in this area can be considered advantageous. This is because the U-profiled petiole is “fixed” in the most favorable position in terms of bending, i.e., with the opening of the U against the acting bending force. Wolff-Vorbeck et al. [34] have shown that, with an increasing size of the groove, the twist-to bend ratio increases because of an increase of the flexural rigidity and a simultaneous decrease of the torsional rigidity. Higher torsional flexibility enables lamina attached to their basal margin to reorient, streamline and thus reduce the wind-induced drag [23,26]. Similar to the U-profile of the petioles of *H. tardiana*, the monocotyledonous banana (*Musa textilis*) has U-profiled petioles, which have a 45 to 100 times higher flexural rigidity than torsional rigidity [24]. Regardless of the particular shape of the petiole, all petioles show a taper in the apical direction, i.e., a gradual change of dimension in terms of the decrease of cross-sectional area.

In summary, irrespective of whether the plant is monocotyledonous or dicotyledonous, the presence of a groove or U-profile on the adaxial side is mainly found in petioles with a 2D-configuration. This shape is advantageous for withstanding high bending forces and for reducing wind-induced drag by (torsional) reorientation. No dependency has been found between the tapering modes and the body plans of the monocotyledons or dicotyledons or the spatial arrangement as 2D- or 3D-configurations.

### 3.3. Gradual Change of Size

The gradient of dimensions can be measured by the change of the cross-sectional area *A* and the variables derived from it, namely the axial second moment of area *I* and the polar second moment of area *J* from the petiolar base to its top. The variables *A*, *I*, and *J* of all samples show a linear fit in the apical petiole and an exponential increase in the petiole-lamina transition zone. However, differences are apparent in the slope *a* and the exponential growth constant *b* of these variables. With the exception of *C. bicolor*, the linear fit of the cross-sectional area reveals (very) small positive or negative values of the slope *a_A_petiole_*, indicating that almost no increase or decrease occurs. However, the different tapering modes *α_petiole_* with various outer shapes (envelopes) influence the linear fit, such as a convex shape (0<α<1; second order paraboloid of revolution), a concave shape (α>1; hyperboloid of revolution), or a straight shape with taper (α≈1; circular cone), or a straight shape without taper (α=0; circular cylinder). The dicotyledonous species studied have higher exponential growth constants *b* for the variables *A*, *I*, and *J* than the monocotyledonous species indicating that their body plan allows them to increase the lamina region more rapidly in the transition zone. This can be explained by a more robust body plan, perhaps based on their internal structuring in terms of the arrangement and merging of vascular bundles, a major strengthening tissue of foliage leaves (see µCT data, Figure 2). The assumption that strengthening tissues have a strong influence on the growth constant *b* is supported by the results in *H. alternata*. This species has the highest *b*-values and is also the only species with a mechanically important hypodermal collenchyma layer.

In summary, all petioles show an approximately linear fit for the variables *A*, *I*, and *J*, but without considerable increase or decrease, with the exception of *C. bicolor*, which shows a slight increase. In contrast, all petiole–lamina transition zones are characterized by an exponential increase of the variables *A*, *I*, and *J*. The change from a linear to an exponential fit is a characteristic that applies to all samples regardless of the body plan or the spatial arrangements of petiole and lamina.

### 3.4. Gradual Change of Tissues Arrangement

Dependent on the atactostele of the monotyledons, the eustele of the dicotyledons and the spatial arrangement of the petiole and lamina with a 2D- or 3D-configuration, similarities and dissimilarities of the arrangement and orientation of the vascular tissues are likely, especially in the transition zone. The petiole of *H. tardiana* shows a parallel venation similar to the lamina, whereas *C. bicolor* reveals a typical atactostele. The venation of *H. alternata* und *P. peperomioides* is typically eustele. In the 2D transition zones, the main part of the vascular bundles runs straight and parallel from the apical petiole into the midrib, with only a few bundles splitting off and entering the basal lamina portions. In the 3D transition zones, the vascular bundles split at the beginning of the transition zone and run in all directions into the lamina. However, individual bundles of the studied monocotyledons are separate, whereas the vascular bundles of the investigated dicotyledons form merged strands. A vascular strand arrangement in the 3D transition zone, comparable with that of the dicotyledonous *P. peperomioides*, has previously been described for the peltate leaves of the dicotyledonous *Malva neglecta* [2], *Kingdonia uniflora* [3], and *Tropaeolum majus* [13]. Another aspect common to all petiole–lamina transition zones is the increase in the number of vascular bundles in the transition zone compared with the apical petiole. The bundles split in order to supply the increasing lamina regions [35]. In all four species studied, this increase is accompanied by a decrease in the area fraction of the vascular tissue *AF_v_*. This can be explained on the basis that the total area of vascular tissue remains almost constant across the transition zones, whereas the total cross-sectional area of the foliage leaf increases, leading to a relative decrease in the area fraction of the vascular tissue. In addition, this transition from thicker parallel bundles to a multitude of thin vascular bundles can also be seen as a gradient of dimensions that allows for load sharing and, thus, a more resilient connection between the petiole and lamina [6,21,26].

In summary, the course of the vascular bundles in the petiole depends on the body plan of the plant as a monocotyledon or dicotyledon. In contrast, the course of the vascular bundles in the transition zone is more dependent on the spatial arrangement than on the body plan. In the 2D transition zones, the vascular bundles run straight and parallel from the apical petiole into the midrib. In contrast, all vascular bundles in the 3D transition zones initially run in the middle of the cross-section and, from there, some groups of vascular bundles curve towards the lamina.

## 4. Materials and Methods

### 4.1. Plant Material

Plants of *H. tardiana*, *C. bicolor*, *H. alternata* and *P. peperomioides* were cultivated in the greenhouse of the Botanic Garden of the University of Freiburg, Germany. The selection criteria for the four species were: (1) two monocotyledonous species and two dicotyledonous species with (2) stalked leaves exhibiting 2D- and 3D-configuration, (3) herbaceous and (4) perennial plants that (5) are easy to cultivate to provide sufficient experimental material. The restriction to herbaceous plants resulted from the limited variety of stalked monocotyledonous species and was intended to allow for better comparability. Leaves of all four plant species were cut at the base of the petiole with a scalpel. One leaf per species was photographed with a Lumix DMC-FZ1000 camera (Panasonic, Kadoma Osaka, Japan; Figure 3a, Figure 4a, Figure 5a and Figure 6a).

### 4.2. Geometry, Size and Shape

The diameters of 25 petioles per species were recorded every 1 cm for *H. tardiana* and *P. peperomioides*, every 3 cm for *C. bicolor*, and every 0.5 cm for *H. alternata*, because of the different lengths of the petioles of the individual species. At each point, the diameter was measured in the lateral direction $d_{l}$ and in the adaxial-abaxial direction $d_{a}$ with a caliper (accuracy ± 0.05 mm) with reference to the lamina. The aspect ratio $AR_{petiole}$ of each petiole sample was calculated by:

(1) $$AR_{petiole}=\frac{d_{l}}{d_{a}},$$

In addition, the tapering mode $\alpha_{petiole}$ was calculated for the 25 petioles per species by using the method described by Caliaro et al. [36]. The tapering mode describes whether the shape of the petiole resembles more a circular cylinder (α=0), a second order paraboloid of revolution (α=0.5), a circular cone (α=1), or a hyperboloid of revolution (α=1.5).

Each image of a transverse thin-section (see Section 4.4) was rotated such that the abaxial side of the section was oriented downwards. The serial transverse sections were the basis for the classification into geometrical categories. Furthermore, in these images, the entire cross-sectional area was masked with the “Threshold” function of Fiji [37] (ImageJ Version 1.52p). The masked areas A together with the axial second moments of area I and the polar second moments of area J were calculated using the function “Slice Geometry” of the BoneJ plugin [38] for Fiji.

The data for A, I, and J were plotted with Microsoft Excel 2016 (Microsoft, Redmond, WA, USA) and the linear and exponential regressions were performed using the built-in functions. The linear slopes a and exponential growth constants b were determined via the given regression equations for the linear (Equation (2)) and the exponential (Equation (3)) regressions:

(2) y(x)=a·x+m,

(3) $$y\left(x\right)=n\cdot e^{b\cdot x},$$

with m being the intercept and n the initial value.

An additional detailed analysis in 100 µm steps was carried out with a single sample of each species. The vascular tissue of each transverse section was masked using GIMP (Version 2.10.6). The number of vascular bundles and the area of the vascular tissue was calculated via the “Analyze Particles” function of Fiji. The percentage area fraction of the vascular tissue $AF_{v}$ was calculated by:

(4) $$AF_{v}\left(\%\right)=\frac{A_{v}}{A_{t}}\cdot 100,$$

where $A_{v}$ is the area of the vascular tissue and $A_{t}$ the total cross-sectional area.

### 4.3. µCT Scanning

One petiole–lamina transition zone sample (see Section 4.4) of each of the four species was critical point dried in acetone (CPD 030, BAL-TEC AG, Balzers, Liechtenstein) and scanned using a high-resolution µCT (Skyscan 1272, Bruker, Kontich, Belgium). The 360° scans were performed with rotation steps of 0.6°, an image resolution of 2452 × 1640, a source voltage of 50 kV without filter, a source current of 200 µA, frame averaging over 3 frames and a random movement correction of 10. Because of the differing sample dimensions, various scan resolutions were used: 6 µm for *H. tardiana*, 3.7 µm for *C. bicolor*, 5.5 µm for *H. alternata*, and 5 µm for *P. peperomioides*. The data were reconstructed in NRecon (Version 1.6.10.1, Bruker, Kontich, Belgium) with a beam hardening correction of 25 %, a ring artefact correction of 5 and smoothing set to 4 for *H. tardiana* and *H. alternata* and to 3 for *C. bicolor* and *P. peperomioides* by using a Gaussian smoothing kernel of 2. The vascular tissues of each sample were segmented and visualized using Avizo 2019.2 (Thermo Fisher Scientific, Waltham, MA, USA). The videos can be found in the Supplementary Materials Videos S1–S4.

### 4.4. Sample Preparation for Histological Studies

Before thin-sectioning, the petiole, petiole–lamina transition zone, and lamina of the leaves were identified for the collection of the respective samples. Samples of the transition zone consisted of the apical 5 mm of the petiole together with a square of 1 cm side length from the lamina. The remaining petiole length was divided into three equal parts that corresponded to the basal, middle, and apical section of the petiole. Prior to thin-sectioning on a rotatory cryotome (MEV, SLEE medical, Mainz, Germany), the samples were frozen on a metal sample holder by using a specific freezing solution (Tissue-Tek O.C.T. Compound, Sakura Finetek Japan Co., Tokyo, Japan). Transverse thin-sections with a thickness of 100 µm were prepared from the basal, middle, and apical part of all petiole samples. Serial transverse thin-sections of the transition zone with a thickness of 200 µm were prepared for five samples of each species. For better anatomical resolution of the transition zone, a sixth sample of each species was serially thin-sectioned at a thickness of 100 µm. Sections of the petiole samples were stained with 0.05% *w*/*v* toluidine blue O [39] for 8 min and afterwards rinsed in distilled water for 8 min. Toluidine blue O (TBO) stains lignified tissue blue to dark violet, while non-lignified tissue is stained red-purple. The 100 µm serial thin-sections of the transition zone were stained with 0.1% *w*/*v* aqueous acridine orange for 8 min and rinsed in distilled water for 8 min. Acridine orange (ACO) stains lignified tissue bright yellow/orange, whereas non-lignified tissue is stained dark brown/red. The 200 µm thin-sections of the transition zones remained unstained. Images of all sections were recorded via a microscope (BX61, Olympus, Tokyo, Japan) equipped with a USB camera (DP71, Olympus, Tokyo, Japan).

### 4.5. Statistics

Raw data are provided in the Supplementary Materials Table S1. The software GNU R 3.6.2 was used for statistical analyses [40]. Parametric data are represented by mean values ± one standard deviation, whereas non-parametric data are shown as median values with respective interquartile ranges (IQR). To determine significant differences of the measured variables between the species and between the petiole and transition zone of each species, Kruskal–Wallis tests were performed together with Wilcoxon signed-rank post hoc tests (with p-value adjustments according to Holm [41]) for paired data and with Mann–Whitney–U post hoc tests (with p-value adjustments according to Holm [41]) for unpaired data, after testing for normal distribution (Shapiro–Wilk test) and for homoscedasticity of the variances (Levene test). For all statistical tests, we employed an alpha level of 5%.

## 5. Conclusions

Although many detailed studies on the morphology, anatomy, and biomechanics of both the petiole and lamina of foliage leaves have been published, the transition zone between them has mostly been ignored. Those scientists who have studied the petiole–lamina transition zone have assigned a variety of terms to this area, depending on their scientific discipline and the underlying scientific question. The transition zone is interesting because, by superimposing various gradients, it creates a damage-resistant transition between the petiole and the lamina, which often differs considerably in geometry, shape, and size. The objective of this study has been to analyze similarities and dissimilarities in the geometry, shape, and size of the petiole and the petiole-lamina transition zone in order to objectively distinguish and characterize the transition zone with at least one key criterion. Our comparative study of four species has included the body plans of monocotyledons and dicotyledons and the various spatial arrangements of petioles and lamina in terms of peltate leaves (3D-configuration) or leaves for which the petiole is marginally attached to the lamina (2D-configuration). Some characteristics are dominated by the body plan (course of vascular tissues in the petiole) and others by the spatial arrangement (change of geometries and course of vascular bundles in the transition zone). However, all the examined samples demonstrate that the investigated transition zones are defined by one key criterion and thereby differ from the petiole and lamina, namely the exponential increase of the cross-sectional area and the axial and polar second moments of area. In conclusion, a variety of terms are used in the literature to describe and characterize the area between petiole and lamina. Nevertheless, to emphasize the 3D nature and integration of multiple gradients of geometry, shape and size, we prefer the term “petiole–lamina transition zone”.

## Figures and Tables

**Table 1:** Compilation of the different terms that can be found in the literature and that are used to describe the transition between the petiole and lamina.

Description	Plant Species	Scientific Field	Author(s)
- transition from petiole to lamina	various *Salvia* species	ontogeny and morphology	Sewell, 1891 [1]
- point of union of petiole and lamina			
- laminar joint	*Malva neglecta*	diaphototropism and anatomy	Yin, 1938 [2]
- point of attachment of the lamina base to the petiole	*Kingdonia uniflora*	morphology and anatomy	Foster and Arnott, 1960 [3]
- point of junction between the base of the lamina and the petiole			
- petiole–lamina junction			
- petiole–lamina juncture	*Populus deltoides*	ontogeny and anatomy	Isebrands and Larson, 1977 [4]
- point where the petiole entered the leaf	*Populus balsamifera*	functional morphology, anatomy and biomechanics	Roth-Nebelsick et al., 2001 [5]
- junction of petiole to lamina	*Liriodendron tulipifera*	biomechanics, functional morphology and physiology	Niinemets and Fleck, 2002 [6]
- attachment point of lamina to petiole			
- border between petiole and lamina	various *Chlorophytum* species	systematics and morphometry	Poulsen and Nordal, 2005 [7]
- petiole–lamina junction	*Acer rubrum*	physiology and morphology	Sack et al., 2008 [8]
	*Acer saccharum*		
	*Betula alleghaniensis*		
	*Kalmia latifolia*		
	*Quercus rubra*		
	*Viburnum acerifolium*		
	*Viburnum cassinoides*		
- petiole-blade junction	various *Ipomoea* species	ontogeny, anatomy and morphometry	Jones and Kang, 2015 [9]
- lamina petiole junction	various *Pelargonium* species	anatomy and allometry	Ray and Jones, 2018 [10]
- petiole/lamina junction	*Acer saccharum*	morphometry and biomechanics	Louf et al., 2018 [11]
	*Liriodendron tulipifera*		
	*Platanus occidentalis*		
	*Quercus rubra*		
- petiole–lamina transition zone	*Caladium bicolor*	biomimetics	Langer et al., 2019 [12]
- transition zone - transition area	*Colocasia fallax*	functional morphology and biomechanics	Sacher et al., 2019 [13]
	*Tropaeolum majus*		
- junction of the blade and the petiole	various *Phyllostachys* and *Pleioblastus* species	allometry and morphometry	Huang et al., 2019 [14]
- point of attachment of leaf lamina and petiole	*Begonia maguniana*	systematics and morphology	Wilson et al., 2019 [15]
- petiole/blade junction - junction of the petiole and blade	various *Schismatoglottis* species	systematics and morphology	Yeng et al., 2019 [16]
- intersection point of the leaf blade and the petiole - intersection point between the leaf blade and petiole	*Acer saccharinum*	functional morphology and biomechanics	Ginebra-Solanellas et al., 2020 [17]
	*Quercus gambelii*		
	*Ulmus pumila*		
- petiole–lamina junction	various species with peltate leaves	functional morphology, systematics	Wunnenberg et al., 2021 [18]
- petiole–lamina transition zone	*Caladium bicolor*	functional morphology, biomimetics	this study
	*Hemigraphis alternata*		
	*Hosta x tardiana* ‘El Niño’		
	*Pilea peperomioides*		

**Table 2:** A list of all analyzed variables and a brief description of each.

Variable	Description
*AR*	Aspect ratio of the diameters in lateral (*d_l_*) and adaxial-abaxial (*d_a_*) direction of the petiole
α	Tapering mode of the petiole
*A*	Cross-sectional area of the transverse section
*I*	Axial second moment of area of the transverse section
*J*	Polar second moment of area of the transverse section
*I/J*	Ratio of axial and polar second moments of area
*a*	Slope of the linear fit calculated for the cross-sectional area (*A*) or the axial second moment of area (*I*) or the polar second moment of area (*J*)
*b*	Growth constant of the exponential fit calculated for the cross-sectional area (*A*) or the axial second moment of area (*I*) or the polar second moment of area (*J*)
*AF_v_*	Area fraction of vascular tissue in relation to the total cross-sectional area (*A*)

**Table 3:** Data of geometry, shape, and size of petioles and transition zones of the four model plants *Hosta x tardiana* ‘El Niño’, *Caladium bicolor*, *Hemigraphis alternata*, and *Pilea peperomioides*.

		Monocotyledons		Dicotyledons		
	Configuration	2D	3D	2D	3D	
	**Species**	***Hosta x*** ***tardiana***	***Caladium*** ***bicolor***	***Hemigraphis*** ***alternata***	***Pilea*** ***peperomioides***	
**Variable**	**Description**	**Median (IQR)**	**Median (IQR)**	**Median (IQR)**	**Median** **(IQR)**	***n***
**Geometry**						
*Geometry change*	Change of the cross-sectional geometry from petiole to transition zone	U-profile → V-profile	circular → curvilinear triangle → triangle with lobes	elliptic → circular	circular → elliptic → lobbed	
**Shape**						
*AR_petiole_* [-]	Aspect ratio of the petiole	1.13 (0.13)	0.95 (0.09)	1.22 (0.09)	1.05 (0.08)	25
*α_petiole_* [-]	Tapering mode of the petiole	1.47 (0.40)	0.91 (0.15)	1.36 (0.57)	1.18 (0.54)	25
*I / J_petiole_* [-]	Ratio of axial and polar second moment of area of the petiole	0.33 (0.05)	0.53 (0.05)	0.40 (0.03)	0.47 (0.04)	25
*I / J_transition_* [-]	Ratio of axial and polar second moment of area of the transition zone	0.45 (0.14)	0.60 (0.13)	0.37 (0.24)	0.42 (0.08)	6
**Size**						
*a_A_petiole_* [mm^2^/mm]	Slope of the linear fit for the cross-sectional area of the apical petiole	0.11 (1.05)	0.65 (0.52)	−0.02 (0.17)	0.12 (0.26)	6
*b_A_transition_* [mm^−1^]	Growth constant of the exponential fit for the cross-sectional area of the transition zone	0.12 (0.11)	0.51 (0.10)	2.73 (1.47)	1.94 (0.80)	6
*a_I_petiole_* [mm^4^/mm]	Slope of the linear fit for the axial second moment of area of the apical petiole	1.60 (4.45)	1.36 (1.42)	−0.01 (0.07)	0.08 (0.27)	6
*b_I_transition_* [mm^−1^]	Growth constant of the exponential fit for the axial second moment of area of the transition zone	0.47 (0.47)	1.35 (0.19)	7.26 (2.11)	3.87 (1.17)	6
*a_J_petiole_* [mm^4^/mm]	Slope of the linear fit for the polar second moment of area of the apical petiole	4.33 (14.99)	2.18 (2.17)	−0.01 (0.17)	0.22 (0.59)	6
*b_J_transition_* [mm^−1^]	Growth constant of the exponential fit for the polar second moment of area of the transition zone	0.47 (0.37)	1.36 (0.16)	10.65 (6.27)	4.18 (1.41)	6

## Data Availability

The data presented in this study are available in the text, tables, figures and Supplementary Materials.

## Supplementary Materials

The following are available online at https://www.mdpi.com/article/10.3390/plants10040774/s1, Video S1: µCT scan of *Hosta x tardiana* ‘El Niño’, Video S2: µCT scan of *Caladium bicolor*. Video S3: µCT scan of *Hemigraphis alternata*, Video S4: µCT scan of *Pilea peperomioides*, Tables S1: Raw data of the various experiments carried out.
