# Supplementary material for: Petiole-Lamina Transition Zone: A Functionally Crucial but Often Overlooked Leaf Trait
Source: Plants (Basel). 2021 Apr 15;10(4):774. doi: 10.3390/plants10040774 (PMC8071152; doi:10.3390/plants10040774)
Supplement: Supplementary file 1 [file plants-10-00774-s001.zip › Langer-et-al-2021_Plants_Supplementary-Materials/S1-Tables_RawData_Langer-et-al-2021.pdf]

Aspect ratio $AR_{petiole}$
of 25 petiole samples

Sample	***Hosta x tardiana* 'El Niño'**	***Caladium bicolor***	***Hemigraphis alternata***	***Pilea peperomioides***
	[-]	[-]	[-]	[-]
1	1.35	0.94	1.22	1.00
2	1.13	0.89	1.24	1.10
3	1.03	0.98	1.22	1.08
4	1.13	0.99	1.24	0.97
5	1.19	0.91	1.40	1.08
6	1.29	0.94	1.15	1.12
7	1.34	0.92	1.44	1.02
8	1.43	1.04	1.23	1.05
9	1.16	0.90	1.12	1.04
10	1.19	0.89	1.14	1.07
11	1.14	0.90	1.23	1.06
12	0.93	1.00	1.15	1.05
13	1.13	0.90	1.21	1.01
14	1.10	1.03	1.25	1.11
15	1.09	0.88	1.18	0.97
16	1.09	1.03	1.30	1.06
17	1.23	1.12	1.12	1.09
18	1.10	1.11	1.12	1.00
19	1.20	0.91	1.31	1.04
20	1.13	0.96	1.20	1.11
21	1.04	0.97	1.17	1.00
22	1.06	0.99	1.24	1.11
23	1.27	0.95	1.25	1.00
24	1.36	0.94	1.16	0.97
25	1.11	1.05	1.17	0.97

Sample	*Hosta x tardiana 'El Niño'*	*Caladium bicolor*	*Hemigraphis alternata*	*Pilea peperomioides*
	[-]	[-]	[-]	[-]
1	1.58	0.79	1.30	1.49
2	1.53	0.76	2.27	0.95
3	1.08	0.82	0.98	1.81
4	1.88	0.84	1.09	0.91
5	1.46	0.91	2.66	1.18
6	1.44	1.11	1.42	0.85
7	1.00	0.87	1.06	1.42
8	1.54	0.84	1.18	0.94
9	1.94	1.18	1.20	1.25
10	1.89	0.90	2.09	1.64
11	2.35	1.04	1.22	1.31
12	1.43	0.96	0.99	1.84
13	0.93	1.06	1.69	0.93
14	1.47	0.72	1.76	1.22
15	1.89	0.88	1.59	1.61
16	1.62	0.99	0.84	0.83
17	1.78	1.10	1.12	1.73
18	1.17	0.99	1.41	1.01
19	1.08	0.93	2.19	0.83
20	1.34	0.91	2.35	1.08
21	1.91	0.83	1.36	1.50
22	1.44	0.91	1.47	1.14
23	1.39	0.75	1.51	1.34
24	1.64	1.39	1.27	1.02
25	1.38	0.85	0.91	1.10

$I/J_{transition}$ of 6 transition zone samples				
**Sample**	***Hosta x tardiana* 'El Niño'**	***Caladium bicolor***	***Hemigraphis alternata***	***Pilea peperomioides***
	[-]	[-]	[-]	[-]
1	0.54	0.54	0.34	0.42
2	0.47	0.65	0.10	0.33
3	0.44	0.49	0.39	0.56
4	0.36	0.55	0.16	0.43
5	0.53	0.74	0.46	0.41
6 (detailed serial section)	0.23	0.68	0.59	0.52

$I/J_{petiole}$ of 25 petiole samples				
**Sample**	***Hosta x tardiana* 'El Niño'**	***Caladium bicolor***	***Hemigraphis alternata***	***Pilea peperomioides***
	[-]	[-]	[-]	[-]
1	0.27	0.56	0.36	0.50
2	0.32	0.56	0.39	0.45
3	0.37	0.51	0.39	0.45
4	0.34	0.50	0.42	0.52
5	0.31	0.54	0.33	0.45
6	0.28	0.52	0.42	0.47
7	0.30	0.54	0.33	0.50
8	0.27	0.49	0.40	0.47
9	0.33	0.56	0.42	0.47
10	0.31	0.55	0.43	0.45
11	0.34	0.58	0.38	0.47
12	0.42	0.48	0.43	0.48
13	0.34	0.58	0.40	0.50
14	0.38	0.51	0.40	0.44
15	0.35	0.56	0.40	0.51
16	0.36	0.49	0.38	0.47
17	0.31	0.44	0.41	0.47
18	0.35	0.51	0.41	0.49
19	0.29	0.55	0.34	0.48
20	0.32	0.52	0.40	0.45
21	0.38	0.51	0.42	0.50
22	0.33	0.53	0.37	0.45
23	0.28	0.54	0.40	0.50
24	0.29	0.54	0.41	0.52
25	0.35	0.47	0.42	0.51

a_A of the apical petiole parts of 6 transition zone samples				
**Sample**	***Hosta x tardiana* 'El Niño'**	***Caladium bicolor***	***Hemigraphis alternata***	***Pilea peperomioides***
	[mm^2/mm]	[mm^2/mm]	[mm^2/mm]	[mm^2/mm]
1	0.07	2.45	-0.09	0.28
2	-0.01	0.83	-0.09	0.12
3	1.59	0.28	0.01	0.42
4	1.37	0.93	-0.04	-0.07
5	-0.06	0.48	0.12	0.13
6 (detailed serial section)	0.14	0.35	0.13	-0.18

a_I of the apical petiole parts of 6 transition zone samples				
**Sample**	***Hosta x tardiana* 'El Niño'**	***Caladium bicolor***	***Hemigraphis alternata***	***Pilea peperomioides***
	[mm^4/mm]	[mm^4/mm]	[mm^4/mm]	[mm^4/mm]
1	0.62	6.60	-0.04	0.39
2	1.47	1.81	-0.08	0.08
3	14.93	0.28	0.00	0.61
4	6.98	1.90	-0.01	0.03
5	1.72	0.91	0.16	0.08
6 (detailed serial section)	1.13	0.31	0.05	-1.58

a_J of the apical petiole parts of 6 transition zone samples				
**Sample**	***Hosta x tardiana* 'El Niño'**	***Caladium bicolor***	***Hemigraphis alternata***	***Pilea peperomioides***
	[mm^4/mm]	[mm^4/mm]	[mm^4/mm]	[mm^4/mm]
1	1.42	12.57	-0.06	0.56
2	4.76	3.07	-0.06	0.12
3	36.73	1.12	0.01	0.90
4	22.83	3.43	-0.03	-0.16
5	3.13	1.30	0.52	0.32
6 (detailed serial section)	3.89	0.93	0.16	-1.14

Explanations:

A = cross-sectional area
I = axial second moment of area
J = polar second moment of area

b_A of the transition zone parts of 6 transition zone samples				
**Sample**	***Hosta x tardiana* 'El Niño'**	***Caladium bicolor***	***Hemigraphis alternata***	***Pilea peperomioides***
	[mm^{-1}]	[mm^{-1}]	[mm^{-1}]	[mm^{-1}]
1	0.08	0.49	6.48	2.14
2	0.29	0.45	3.32	1.74
3	0.12	0.54	2.16	2.67
4	0.22	0.37	3.31	1.74
5	0.12	0.71	1.75	3.33
6 (detailed serial section)	0.06	0.56	0.87	0.58

b_I of the transition zone parts of 6 transition zone samples				
**Sample**	***Hosta x tardiana* 'El Niño'**	***Caladium bicolor***	***Hemigraphis alternata***	***Pilea peperomioides***
	[mm^{-1}]	[mm^{-1}]	[mm^{-1}]	[mm^{-1}]
1	0.29	1.31	19.61	3.83
2	1.26	1.32	9.06	3.59
3	0.45	1.37	7.24	5.13
4	0.95	1.14	7.28	3.90
5	0.48	1.56	6.25	6.45
6 (detailed serial section)	0.33	1.55	3.68	1.30

b_J of the transition zone parts of 6 transition zone samples				
**Sample**	***Hosta x tardiana* 'El Niño'**	***Caladium bicolor***	***Hemigraphis alternata***	***Pilea peperomioides***
	[mm^{-1}]	[mm^{-1}]	[mm^{-1}]	[mm^{-1}]
1	0.32	1.32	22.73	4.42
2	1.02	1.24	12.38	3.74
3	0.49	1.39	8.92	5.45
4	0.80	1.17	13.40	3.94
5	0.45	1.67	6.20	8.29
6 (detailed serial section)	0.31	1.43	3.12	1.27

Explanations:

A = cross-sectional area
I = axial second moment of area
J = polar second moment of area

Sample 1			
**Section height**	**Cross-sectional area *A***	**Axial second moment of area *I***	**Polar second moment of area *J***
[mm]	[mm^2]	[mm^4]	[mm^4]
0.0	8.23	8.05	19.44
0.2	9.41	11.02	23.42
0.4	9.16	9.88	22.70
0.6	9.45	11.13	23.58
0.8	8.93	9.01	22.45
1.0	8.93	9.20	22.26
1.2	8.95	9.96	21.54
1.4	8.80	8.50	22.18
1.6	8.77	8.85	22.07
1.8	9.10	9.71	23.52
2.0	8.99	9.51	23.34
2.2	9.07	10.28	23.71
2.4	9.19	10.63	23.30
2.6	9.19	10.14	24.54
2.8	9.20	10.18	24.20
3.0	9.09	10.33	23.93
3.2	9.23	10.93	24.35
3.4	9.13	10.01	24.94
3.6	9.17	10.35	24.87
3.8	9.14	10.14	25.29
4.0	9.48	11.96	25.98
4.2	9.18	10.67	25.74
4.4	8.94	10.44	24.77
4.6	9.01	10.54	25.89
4.8	9.29	11.74	27.23
5.0	9.41	11.75	28.95
5.2	9.28	10.37	27.47
5.4	9.55	13.31	30.23
5.6	9.05	10.45	25.70
5.8	9.38	11.95	28.95
6.0	9.49	13.50	30.73
6.2	9.19	10.94	28.53
6.4	9.62	14.32	32.10
6.6	9.29	12.48	29.16
6.8	9.56	15.71	33.58
7.0	9.37	14.49	30.99
7.2	9.65	14.86	31.82
7.4	11.42	27.82	55.53
7.6	10.43	21.08	37.74
7.8	10.69	24.84	43.06
8.0	10.75	24.81	42.60
8.2	10.98	27.53	50.44
8.4	9.50	16.59	34.02
8.6	11.62	28.75	67.49

Sample 1 (continued)			
**Section height**	**Cross-sectional area *A***	**Axial second moment of area *I***	**Polar second moment of area *J***
[mm]	[mm^2]	[mm^4]	[mm^4]
8.8	11.49	31.08	58.21
9.0	10.64	33.03	49.60
9.2	12.62	38.49	78.19
9.4	12.50	40.03	60.33
9.6	11.00	33.64	57.72
9.8	13.54	41.51	130.41

Sample 2			
**Section height**	**Cross-sectional area *A***	**Axial second moment of area *I***	**Polar second moment of area *J***
[mm]	[mm^2]	[mm^4]	[mm^4]
0.0	15.16	19.57	60.00
0.2	15.00	18.92	58.46
0.4	15.41	18.90	62.99
0.6	15.38	21.01	63.93
0.8	15.28	22.05	67.24
1.0	15.41	22.14	66.10
1.2	15.25	21.15	65.17
1.4	14.99	20.04	61.32
1.6	15.37	23.63	70.88
1.8	15.17	22.95	70.37
2.0	15.31	22.33	73.05
2.2	15.24	22.63	71.31
2.4	14.84	21.05	65.60
2.6	14.98	20.89	68.55
2.8	14.61	21.20	67.26
3.0	15.06	22.49	71.48
3.2	15.51	22.94	78.67
3.4	15.27	24.20	75.33
3.6	14.65	20.81	67.09
3.8	15.22	26.00	77.35
4.0	15.95	31.64	93.52
4.2	16.15	33.39	99.87
4.4	17.33	63.55	144.35
4.6	17.75	80.35	155.51
4.8	18.71	81.54	173.17
5.0	18.76	93.12	187.26
5.2	22.82	155.68	395.24
5.4	22.53	183.42	321.16

Sample 3			
Section height	Cross-sectional area *A*	Axial second moment of area *I*	Polar second moment of area *J*
[mm]	[mm^2]	[mm^4]	[mm^4]
0.0	17.47	31.41	72.58
0.2	15.22	20.41	50.87
0.4	14.06	14.90	42.92
0.6	18.87	37.04	124.53
0.8	17.94	30.58	91.19
1.0	17.91	32.95	94.79
1.2	16.13	22.97	65.64
1.4	15.54	19.92	58.19
1.6	19.51	53.90	139.79
1.8	19.14	59.17	130.71
2.0	19.28	43.10	124.86
2.2	21.39	79.02	189.14
2.4	19.61	51.81	144.09
2.6	18.83	46.13	124.35
2.8	17.44	44.95	106.67
3.0	21.24	102.93	234.23
3.2	21.70	108.27	246.64
3.4	22.14	125.44	240.71
3.6	19.81	100.10	185.04
3.8	23.68	113.46	386.81
4.0	22.52	122.55	279.94
4.2	23.89	142.55	377.05
4.4	23.73	137.65	377.38
4.6	22.68	158.84	304.02

Sample 4			
Section height	Cross-sectional area *A*	Axial second moment of area *I*	Polar second moment of area *J*
[mm]	[mm^2]	[mm^4]	[mm^4]
0.0	11.20	11.87	38.93
0.2	9.74	11.29	27.48
0.4	9.42	9.09	24.02
0.6	7.99	6.24	18.87
0.8	9.12	8.67	26.06
1.0	9.56	8.00	26.48
1.2	9.22	8.81	28.96
1.4	9.70	11.27	31.95
1.6	11.95	18.91	58.74
1.8	11.69	19.81	58.54
2.0	12.96	21.56	75.18
2.2	11.05	18.86	54.04
2.4	13.24	28.83	89.58

Sample 4 (continued)			
**Section height**	**Cross-sectional area *A***	**Axial second moment of area *I***	**Polar second moment of area *J***
[mm]	[mm^2]	[mm^4]	[mm^4]
2.6	12.48	25.89	82.42
2.8	15.13	62.56	154.54
3.0	14.68	45.36	142.80
3.2	16.12	70.44	196.92
3.4	15.08	47.01	166.44
3.6	16.72	90.26	238.17
3.8	17.50	117.07	263.27

Sample 5			
**Section height**	**Cross-sectional area *A***	**Axial second moment of area *I***	**Polar second moment of area *J***
[mm]	[mm^2]	[mm^4]	[mm^4]
0.0	15.63	20.80	54.71
0.2	15.01	20.13	53.65
0.4	14.80	21.37	51.52
0.6	14.28	17.71	46.12
0.8	14.92	21.89	53.95
1.0	15.21	24.23	58.02
1.2	14.90	22.42	60.89
1.4	13.48	17.22	44.46
1.6	14.41	19.68	52.90
1.8	12.78	15.97	42.29
2.0	14.13	18.76	53.67
2.2	14.32	21.13	53.47
2.4	14.18	20.83	49.38
2.6	12.93	23.51	43.02
2.8	14.45	23.65	71.97
3.0	12.90	20.71	39.84
3.2	14.06	20.15	47.48
3.4	12.15	13.87	43.14
3.6	14.38	23.24	58.73
3.8	12.41	21.97	50.29
4.0	11.56	18.37	48.69
4.2	14.34	22.63	57.98
4.4	14.59	22.85	82.60
4.6	15.43	27.86	70.02
4.8	14.87	19.46	58.32
5.0	15.18	29.42	63.95
5.2	13.95	26.54	65.56
5.4	16.82	43.87	95.52
5.6	13.55	25.04	55.91
5.8	12.52	23.16	45.64

Sample 5 (continued)			
Section height	Cross-sectional area *A*	Axial second moment of area *I*	Polar second moment of area *J*
[mm]	[mm^2]	[mm^4]	[mm^4]
6.0	15.40	36.82	80.04
6.2	13.84	39.10	64.74
6.4	17.03	56.76	106.77
6.6	16.76	54.93	106.45
6.8	16.95	56.76	111.10
7.0	16.35	59.40	112.99
7.2	17.75	72.40	124.88
7.4	17.87	76.25	138.76
7.6	18.63	85.58	145.97
7.8	17.24	81.08	155.69
8.0	18.59	87.79	168.31
8.2	19.01	94.54	177.99
8.4	17.00	69.85	138.12
8.6	20.16	129.27	201.25
8.8	21.03	149.44	237.95
9.0	21.09	150.62	278.02
9.2	22.96	196.42	383.24
9.4	24.00	296.87	426.39

Sample 6 (detailed serial section)					
Section height	Cross-sectional area *A*	Axial second moment of area *I*	Polar second moment of area *J*	Number of vascular bundles	Area fraction of the vascular bundles AF_v
[mm]	[mm^2]	[mm^4]	[mm^4]	[-]	[%]
0.0	6.70	4.81	16.50	18	8.30
0.4	5.81	3.06	10.93	14	8.75
0.5	6.14	3.86	12.64	15	7.88
0.6	6.48	4.06	14.11	16	7.79
1.0	6.11	3.61	14.36	18	8.16
1.1	6.53	4.67	17.97	17	7.87
1.2	5.88	3.61	11.81	14	7.08
1.3	6.04	3.96	14.05	17	8.07
1.4	6.06	3.58	14.55	16	7.95
1.5	6.31	4.59	17.57	20	8.23
1.6	6.32	4.41	17.82	21	7.88
1.7	5.90	3.73	13.50	17	7.98
1.8	6.30	4.89	18.57	19	8.77
1.9	6.36	5.34	18.81	19	8.45
2.2	6.16	4.92	16.26	20	8.31
2.3	6.38	5.47	19.72	20	8.20
2.4	6.06	4.68	16.34	19	8.16
2.5	6.11	4.87	16.76	17	8.52
2.6	6.38	5.75	20.22	17	7.67

Sample 6 (detailed serial section) (continued)					
**Section height**	**Cross-sectional area *A***	**Axial second moment of area *I***	**Polar second moment of area *J***	**Number of vascular bundles**	**Area fraction of the vascular bundles AF_v**
[mm]	[mm^2]	[mm^4]	[mm^4]	[-]	[%]
2.8	6.47	6.07	21.50	17	7.14
2.9	6.24	5.07	18.44	16	6.89
3.1	6.26	5.45	19.00	16	7.40
3.2	6.24	4.74	19.16	16	7.47
3.4	6.60	6.01	24.46	20	8.01
3.5	6.59	7.11	25.17	19	9.00
3.7	6.63	6.84	25.65	20	7.74
3.8	6.52	8.24	21.83	17	8.43
3.9	6.77	6.85	28.26	18	7.34
4.0	6.55	7.88	24.28	17	7.31
4.1	6.85	5.87	31.90	17	7.48
4.2	6.79	8.68	28.66	18	6.88
4.4	6.68	9.23	25.30	18	7.08
4.5	7.02	9.34	33.86	19	6.87
4.6	6.93	7.62	33.32	19	7.77
4.9	7.12	10.09	37.55	20	7.12
5.0	7.23	12.38	39.20	21	7.61
5.1	7.23	13.28	39.22	20	6.73
5.2	7.37	7.85	44.64	18	6.45
5.4	7.50	10.56	45.45	17	6.47
5.5	7.57	13.77	46.09	18	6.72
5.7	7.53	11.59	49.38	19	6.72
5.8	7.69	13.94	51.59	20	6.91
5.9	7.68	13.48	52.43	21	7.47
6.0	7.71	11.65	56.35	20	7.26
6.4	7.95	11.93	65.12	20	6.38
6.5	8.14	13.39	67.39	20	5.67
6.7	7.92	27.45	54.47	21	6.25
6.9	8.35	8.89	82.39	24	6.58
7.0	8.17	13.57	77.40	21	8.28
7.1	8.23	15.41	81.06	20	6.67
7.2	8.27	27.47	72.47	20	5.94
7.3	8.25	12.39	84.86	20	6.05
7.4	8.35	19.82	87.09	20	6.31
7.5	8.47	23.77	88.55	22	6.67
8.0	8.75	35.17	98.32	22	6.50
8.1	8.68	42.01	89.78	23	6.61

Explanations:

basal end of the sample => section height: 0.0 mm

Sample 1			
**Section height**	**Cross-sectional area *A***	**Axial second moment of area *I***	**Polar second moment of area *J***
[mm]	[mm^2]	[mm^4]	[mm^4]
0.0	11.37	9.39	20.75
0.2	10.73	8.46	18.43
0.4	10.72	8.48	18.41
0.6	10.73	8.63	18.43
0.8	10.98	8.98	19.30
1.0	10.99	8.96	19.34
1.2	11.03	9.16	19.50
1.4	11.20	9.57	20.04
1.6	11.47	10.03	21.04
1.8	11.46	10.00	21.01
2.0	11.74	10.42	22.06
2.2	11.85	10.76	22.47
2.4	12.26	11.42	24.06
2.6	12.86	12.54	26.55
2.8	13.30	14.04	28.36
3.0	14.73	16.90	35.30
3.2	15.71	19.67	40.25
3.4	17.06	23.40	47.48
3.6	18.30	27.25	54.85
3.8	19.63	31.33	63.60
4.0	21.12	38.03	74.63
4.2	21.65	42.00	79.64
4.4	23.33	49.67	94.21
4.6	26.92	71.24	132.11
4.8	27.82	78.65	144.80
5.0	31.54	106.36	197.36
5.2	34.77	139.55	256.18
5.4	41.21	216.32	398.27
5.6	45.08	268.63	503.56
5.8	48.48	328.53	632.52
6.0	47.69	360.04	687.50

Sample 2			
**Section height**	**Cross-sectional area *A***	**Axial second moment of area *I***	**Polar second moment of area *J***
[mm]	[mm^2]	[mm^4]	[mm^4]
0.0	8.80	5.60	12.50
0.2	9.65	7.93	14.91
0.4	8.49	5.17	11.57
0.6	8.26	5.16	10.91
0.8	8.38	5.23	11.25
1.0	8.32	5.19	11.10

Sample 2 (continued)			
Section height	Cross-sectional area *A*	Axial second moment of area *I*	Polar second moment of area *J*
[mm]	[mm^2]	[mm^4]	[mm^4]
1.2	8.29	5.10	11.00
1.4	8.28	5.08	10.97
1.6	8.50	5.29	11.58
1.8	8.13	5.26	10.59
2.0	8.38	5.15	11.30
2.2	8.69	5.73	12.11
2.4	8.79	6.10	12.41
2.6	8.87	6.20	12.64
2.8	9.37	6.75	14.11
3.0	10.60	9.60	18.68
3.2	12.35	13.48	25.70
3.4	13.02	15.83	28.88
3.6	13.28	14.68	29.73
3.8	14.97	22.22	38.59
4.0	17.46	32.47	53.46
4.2	19.09	40.66	66.08
4.4	19.50	46.52	71.92
4.6	23.60	74.07	110.60
4.8	26.92	100.50	147.94
5.0	29.57	125.39	185.35
5.2	32.10	158.18	237.79
5.4	32.13	194.58	290.19
5.6	32.89	233.64	359.69
5.8	33.69	265.36	425.51

Sample 3			
Section height	Cross-sectional area *A*	Axial second moment of area *I*	Polar second moment of area *J*
[mm]	[mm^2]	[mm^4]	[mm^4]
0.0	9.92	8.92	16.61
0.2	10.17	7.98	16.51
0.4	10.42	8.64	17.39
0.6	9.91	7.35	15.68
0.8	10.01	7.61	16.04
1.0	9.78	7.27	15.27
1.2	9.60	7.07	14.70
1.4	9.63	6.91	14.83
1.6	9.21	6.53	13.56
1.8	9.36	6.57	14.03
2.0	9.56	6.80	14.68
2.2	9.47	6.62	14.36
2.4	9.49	6.76	14.40

Sample 3 (continued)			
**Section height**	**Cross-sectional area *A***	**Axial second moment of area *I***	**Polar second moment of area *J***
[mm]	[mm^2]	[mm^4]	[mm^4]
2.6	9.28	6.59	13.79
2.8	9.19	6.28	13.53
3.0	9.22	6.18	13.65
3.2	8.89	5.73	12.70
3.4	8.88	5.55	12.72
3.6	9.30	6.27	13.90
3.8	9.34	6.26	14.03
4.0	9.24	6.13	13.78
4.2	9.59	6.57	14.87
4.4	9.93	7.25	15.87
4.6	10.15	7.24	16.69
4.8	10.67	8.05	18.49
5.0	11.42	9.06	21.34
5.2	12.83	11.75	27.04
5.4	13.91	13.77	31.96
5.6	13.90	13.79	31.93
5.8	19.40	32.10	65.08
6.0	20.67	36.68	74.72
6.2	23.98	52.73	105.27
6.4	25.80	66.13	128.32
6.6	28.43	81.64	164.07
6.8	32.71	117.10	229.03
7.0	36.65	150.01	303.54
7.2	43.31	218.75	458.07
7.4	43.25	227.63	527.47
7.6	47.30	302.12	650.47
7.8	47.80	351.08	762.28

Sample 4			
**Section height**	**Cross-sectional area *A***	**Axial second moment of area *I***	**Polar second moment of area *J***
[mm]	[mm^2]	[mm^4]	[mm^4]
0.0	8.80	5.97	12.38
0.2	9.25	6.01	13.88
0.4	9.42	6.57	14.27
0.6	9.61	6.83	14.85
0.8	9.06	6.46	13.13
1.0	8.68	5.55	12.14
1.2	8.95	6.28	12.85
1.4	8.72	6.06	12.18
1.6	8.98	6.43	12.93
1.8	8.85	6.06	12.54

Sample 4 (continued)			
**Section height**	**Cross-sectional area *A***	**Axial second moment of area *I***	**Polar second moment of area *J***
[mm]	[mm^2]	[mm^4]	[mm^4]
2.0	8.70	5.87	12.13
2.2	8.41	5.43	11.38
2.4	8.84	5.92	12.54
2.6	9.08	6.46	13.21
2.8	9.07	6.43	13.18
3.0	9.94	7.86	15.87
3.2	10.19	8.19	16.80
3.4	10.42	8.46	17.57
3.6	12.05	11.36	23.94
3.8	12.63	12.71	26.28
4.0	13.40	15.26	29.53
4.2	14.13	18.05	32.94
4.4	14.74	19.57	35.98
4.6	15.89	23.53	42.38
4.8	17.75	30.17	54.60
5.0	19.24	37.05	65.46
5.2	21.12	46.28	81.79
5.4	24.69	67.67	119.54
5.6	26.57	80.41	141.58
5.8	30.68	110.08	198.24
6.0	32.01	138.37	252.41
6.2	32.49	165.70	308.18
6.4	33.55	207.90	398.78
6.6	34.20	233.21	442.33
6.8	36.01	290.02	567.27
7.0	36.55	320.49	648.70

Sample 5			
**Section height**	**Cross-sectional area *A***	**Axial second moment of area *I***	**Polar second moment of area *J***
[mm]	[mm^2]	[mm^4]	[mm^4]
0.0	6.13	3.01	6.00
0.2	6.62	3.56	6.99
0.4	6.15	3.04	6.02
0.6	6.05	2.99	5.83
0.8	5.94	2.77	5.62
1.0	5.85	2.70	5.45
1.2	5.63	2.44	5.06
1.4	5.76	2.58	5.29
1.6	5.55	2.38	4.90
1.8	5.69	2.54	5.16
2.0	5.69	2.51	5.16

Sample 5 (continued)			
**Section height**	**Cross-sectional area *A***	**Axial second moment of area *I***	**Polar second moment of area *J***
[mm]	[mm^2]	[mm^4]	[mm^4]
2.2	5.75	2.62	5.28
2.4	5.83	2.69	5.42
2.6	5.86	2.70	5.47
2.8	5.90	2.73	5.56
3.0	6.08	2.96	5.90
3.2	6.30	3.19	6.35
3.4	6.49	3.48	6.75
3.6	7.15	4.47	8.24
3.8	8.48	6.86	11.89
4.0	10.17	11.90	18.10
4.2	11.04	16.51	23.11
4.4	14.04	32.06	41.45
4.6	17.61	58.36	72.39
4.8	18.74	63.03	79.70
5.0	24.53	111.74	143.59
5.2	28.22	134.60	182.40
5.4	31.12	149.92	211.85
5.6	29.13	160.79	250.54
5.8	36.68	274.21	424.19

Sample 6 (detailed serial section)					
**Section height**	**Cross-sectional area *A***	**Axial second moment of area *I***	**Polar second moment of area *J***	**Number of vascular bundles**	**Area fraction of the vascular bundles AF_v**
[mm]	[mm^2]	[mm^4]	[mm^4]	[-]	[%]
0.0	6.74	3.20	7.31	75.00	5.38
0.1	6.89	3.39	7.64	74.00	3.96
0.2	6.87	3.34	7.61	72.00	3.27
0.3	6.89	3.36	7.64	74.00	3.58
0.4	6.95	3.46	7.79	74.00	3.37
0.5	6.90	3.39	7.66	75.00	3.79
0.6	6.94	3.42	7.76	77.00	3.76
0.7	6.78	3.23	7.42	75.00	4.36
0.8	6.97	3.48	7.82	74.00	4.29
0.9	6.81	3.29	7.47	76.00	4.32
1.0	6.87	3.34	7.61	79.00	4.59
1.1	6.84	3.33	7.52	73.00	4.66
1.2	6.86	3.30	7.59	80.00	4.06
1.3	6.83	3.33	7.52	77.00	4.72
1.4	6.92	3.40	7.72	78.00	5.28
1.5	6.90	3.36	7.67	73.00	5.22
1.6	6.71	3.10	7.28	76.00	5.22
1.7	6.94	3.37	7.77	73.00	4.75

Sample 6 (detailed serial section) (continued)					
**Section height**	**Cross-sectional area *A***	**Axial second moment of area *I***	**Polar second moment of area *J***	**Number of vascular bundles**	**Area fraction of the vascular bundles AF_v**
[mm]	[mm^2]	[mm^4]	[mm^4]	[-]	[%]
1.8	6.64	2.89	7.20	77.00	4.49
1.9	7.05	3.01	8.26	71.00	4.88
2.0	6.87	2.96	7.79	78.00	4.99
2.2	6.95	3.03	7.97	76.00	5.41
2.3	6.89	2.97	7.84	71.00	5.89
2.4	6.80	2.91	7.62	75.00	5.83
2.5	6.97	3.02	8.03	75.00	5.52
2.6	7.07	3.10	8.29	72.00	5.24
2.7	7.03	3.04	8.19	72.00	5.45
2.8	7.08	3.15	8.26	71.00	6.02
2.9	7.10	3.13	8.32	72.00	5.93
3.0	7.04	3.12	8.16	73.00	5.71
3.1	7.04	3.08	8.19	70.00	5.60
3.2	7.14	3.06	8.50	69.00	6.00
3.4	7.16	3.16	8.49	69.00	6.00
3.5	7.18	3.26	8.48	69.00	5.78
3.6	7.12	3.18	8.39	67.00	5.69
3.7	7.16	3.20	8.46	68.00	5.53
3.8	7.19	3.25	8.50	66.00	5.60
3.9	7.31	3.38	8.81	66.00	4.89
4.0	7.41	3.47	9.06	68.00	5.02
4.1	7.56	3.60	9.43	68.00	4.95
4.2	7.68	3.74	9.73	71.00	4.99
4.3	7.72	3.82	9.85	67.00	4.58
4.4	7.85	3.94	10.19	69.00	4.77
4.5	8.13	4.24	10.94	69.00	4.50
4.6	8.39	4.55	11.65	71.00	4.38
4.7	8.43	4.74	11.72	69.00	3.99
4.8	8.59	5.11	12.11	68.00	4.07
4.9	9.05	5.77	13.41	68.00	4.34
5.0	9.37	6.35	14.33	69.00	3.79
5.1	9.58	6.78	15.06	70.00	3.74
5.3	10.60	8.76	18.50	73.00	3.05
5.6	16.23	31.84	48.89	86.00	2.85
5.7	17.31	37.50	56.60	89.00	2.63
5.8	18.11	42.08	63.13	90.00	2.68
5.9	19.50	50.83	74.80	97.00	2.82
6.0	20.55	56.89	84.02	99.00	2.79
6.2	22.90	74.18	107.92	97.00	2.76
6.3	24.68	85.85	125.38	99.00	3.92
6.4	25.18	93.69	135.33	108.00	2.87
6.5	27.03	114.91	163.94	118.00	2.82
6.6	27.66	117.68	174.27	121.00	2.77
6.7	28.07	136.44	199.60	132.00	3.12

Sample 6 (detailed serial section) (continued)					
**Section height**	**Cross-sectional area *A***	**Axial second moment of area *I***	**Polar second moment of area *J***	**Number of vascular bundles**	**Area fraction of the vascular bundles AF_v**
[mm]	[mm^2]	[mm^4]	[mm^4]	[-]	[%]
6.8	28.77	146.73	219.34	125.00	3.72
6.9	28.88	165.65	245.66	125.00	3.09

Explanations:

basal end of the sample => section height: 0.0 mm

Sample 1			
**Section height**	**Cross-sectional area *A***	**Axial second moment of area *I***	**Polar second moment of area *J***
[mm]	[mm^2]	[mm^4]	[mm^4]
0.0	1.50	0.13	0.40
0.2	2.91	0.52	1.42
0.4	3.07	0.59	1.58
0.6	3.04	0.54	1.58
0.8	3.11	0.68	1.57
1.0	2.65	0.44	1.18
1.2	1.75	0.17	0.54
1.4	1.93	0.22	0.63
1.6	2.83	0.42	1.41
1.8	2.51	0.37	1.08
2.0	1.80	0.18	0.56
2.2	1.92	0.20	0.65
2.4	1.87	0.19	0.60
2.6	2.03	0.22	0.72
2.8	2.19	0.27	0.84
3.0	1.79	0.18	0.55
3.2	2.20	0.25	0.87
3.4	2.65	0.36	1.35
3.6	3.02	0.57	1.61
3.8	3.52	0.91	2.04
4.0	12.87	45.78	191.85

Sample 2			
**Section height**	**Cross-sectional area *A***	**Axial second moment of area *I***	**Polar second moment of area *J***
[mm]	[mm^2]	[mm^4]	[mm^4]
0.0	3.03	0.69	1.50
0.2	3.22	0.78	1.67
0.4	3.07	0.67	1.52
0.6	3.06	0.67	1.51
0.8	2.45	0.40	0.99
1.0	2.85	0.53	1.34
1.2	2.13	0.26	0.79
1.4	2.38	0.34	0.96
1.6	2.76	0.49	1.26
1.8	2.56	0.40	1.10
2.0	2.57	0.37	1.15
2.2	2.85	0.52	1.35
2.4	2.84	0.51	1.32
2.6	2.94	0.55	1.43
2.8	2.78	0.47	1.36
3.0	3.06	0.59	1.55

Sample 2 (continued)			
**Section height**	**Cross-sectional area *A***	**Axial second moment of area *I***	**Polar second moment of area *J***
[mm]	[mm^2]	[mm^4]	[mm^4]
3.2	6.42	4.58	65.56
3.4	11.56	22.11	218.84

Sample 3			
**Section height**	**Cross-sectional area *A***	**Axial second moment of area *I***	**Polar second moment of area *J***
[mm]	[mm^2]	[mm^4]	[mm^4]
0.0	1.96	0.24	0.65
0.2	1.75	0.20	0.51
0.4	1.65	0.17	0.46
0.6	1.98	0.30	0.67
0.8	2.14	0.32	0.75
1.0	1.50	0.15	0.38
1.2	1.09	0.07	0.21
1.4	2.13	0.32	0.79
1.6	1.53	0.17	0.40
1.8	1.82	0.17	0.60
2.0	1.31	0.10	0.29
2.2	2.31	0.38	0.88
2.4	2.01	0.24	0.69
2.6	2.16	0.33	0.76
2.8	1.95	0.28	0.64
3.0	2.14	0.29	0.83
3.2	1.99	0.26	0.66
3.4	1.74	0.20	0.50
3.6	1.20	0.07	0.26
3.8	1.47	0.13	0.39
4.0	1.86	0.18	0.62
4.2	2.08	0.29	0.71
4.4	1.88	0.22	0.60
4.6	2.09	0.29	0.73
4.8	4.97	19.42	46.46
5.0	5.37	28.05	74.51
5.2	8.58	32.16	236.93

Sample 4			
**Section height**	**Cross-sectional area *A***	**Axial second moment of area *I***	**Polar second moment of area *J***
[mm]	[mm^2]	[mm^4]	[mm^4]
0.0	2.10	0.27	0.73

Sample 4 (continued)			
Section height	Cross-sectional area *A*	Axial second moment of area *I*	Polar second moment of area *J*
[mm]	[mm^2]	[mm^4]	[mm^4]
0.2	2.45	0.42	0.97
0.4	2.40	0.40	0.93
0.6	2.34	0.37	0.89
0.8	2.36	0.39	0.91
1.0	2.28	0.34	0.85
1.2	2.19	0.34	0.78
1.4	2.08	0.32	0.70
1.6	2.35	0.37	0.90
1.8	2.13	0.28	0.76
2.0	2.31	0.37	0.87
2.2	2.24	0.33	0.82
2.4	1.96	0.25	0.63
2.6	2.38	0.40	0.92
2.8	2.14	0.32	0.75
3.0	2.13	0.28	0.78
3.2	2.44	0.47	0.97
3.4	2.04	0.27	0.69
3.6	2.05	0.27	0.68
3.8	2.26	0.35	0.84
4.0	2.11	0.31	0.73
4.2	5.48	8.03	51.41
4.4	7.93	5.69	155.70

Sample 5			
Section height	Cross-sectional area *A*	Axial second moment of area *I*	Polar second moment of area *J*
[mm]	[mm^2]	[mm^4]	[mm^4]
0.0	5.17	1.63	4.44
0.2	5.14	1.61	4.42
0.4	5.30	1.77	4.65
0.6	5.26	1.91	4.60
0.8	5.35	1.82	4.72
1.0	5.32	1.97	4.64
1.2	5.41	1.93	4.94
1.4	5.03	1.63	4.23
1.6	5.83	2.13	5.83
1.8	5.17	1.61	4.55
2.0	5.05	1.73	4.20
2.2	5.49	1.91	5.14
2.4	5.35	1.79	4.87
2.6	5.33	1.93	4.79
2.8	5.73	2.14	5.87

Sample 5 (continued)			
**Section height**	**Cross-sectional area *A***	**Axial second moment of area *I***	**Polar second moment of area *J***
[mm]	[mm^2]	[mm^4]	[mm^4]
3.0	5.37	1.95	5.17
3.2	5.82	2.79	7.97
3.4	6.14	3.28	7.83
3.6	9.24	21.44	35.52
3.8	13.15	61.87	141.91
4.0	17.47	148.50	307.17

Sample 6 (detailed serial section)					
**Section height**	**Cross-sectional area *A***	**Axial second moment of area *I***	**Polar second moment of area *J***	**Number of vascular bundles**	**Area fraction of the vascular bundles AF_v**
[mm]	[mm^2]	[mm^4]	[mm^4]	[-]	[%]
0.0	2.35	0.38	0.90	26.00	1.41
0.1	2.35	0.37	0.90	26.00	1.41
0.4	2.35	0.37	0.90	28.00	1.54
0.5	2.38	0.38	0.92	28.00	1.50
0.6	2.33	0.34	0.89	27.00	1.48
0.7	2.31	0.35	0.88	27.00	1.51
0.8	2.36	0.37	0.91	26.00	1.63
0.9	2.31	0.35	0.87	29.00	1.62
1.0	2.38	0.38	0.92	29.00	1.56
1.1	2.39	0.38	0.93	31.00	1.48
1.2	2.38	0.37	0.93	28.00	1.62
1.3	2.28	0.36	0.85	29.00	1.56
1.6	1.94	0.26	0.61	28.00	1.71
1.8	2.31	0.34	0.87	29.00	1.58
1.9	2.45	0.40	0.98	28.00	1.48
2.0	2.37	0.37	0.92	31.00	1.53
2.1	2.38	0.37	0.93	30.00	1.57
2.2	2.44	0.40	0.98	33.00	1.72
2.3	2.39	0.39	0.93	34.00	1.62
2.4	2.43	0.38	0.97	34.00	1.49
2.5	2.43	0.40	0.97	35.00	1.35
2.6	2.52	0.41	1.05	36.00	1.57
2.7	2.44	0.39	0.98	34.00	1.71
2.8	2.43	0.40	0.97	35.00	1.50
3.0	2.52	0.42	1.04	36.00	1.66
3.1	2.47	0.40	1.00	36.00	1.40
3.2	2.41	0.38	0.96	36.00	1.70
3.3	2.49	0.41	1.02	39.00	1.62
3.4	2.51	0.42	1.05	40.00	1.73
3.5	2.53	0.42	1.07	40.00	1.66
3.6	2.53	0.41	1.07	39.00	1.69

Sample 6 (detailed serial section) (continued)					
**Section height**	**Cross-sectional area *A***	**Axial second moment of area *I***	**Polar second moment of area *J***	**Number of vascular bundles**	**Area fraction of the vascular bundles AF_v**
[mm]	[mm^2]	[mm^4]	[mm^4]	[-]	[%]
3.7	2.52	0.40	1.07	38.00	1.58
3.8	2.53	0.42	1.08	37.00	1.66
3.9	2.60	0.44	1.15	41.00	1.55
4.0	2.61	0.44	1.16	38.00	1.62
4.1	2.65	0.45	1.20	41.00	1.57
4.2	2.62	0.43	1.18	42.00	1.62
4.3	2.63	0.45	1.21	45.00	1.52
4.4	2.71	0.48	1.29	43.00	1.66
4.5	2.72	0.47	1.32	43.00	1.57
4.6	2.82	0.52	1.44	42.00	1.54
4.7	2.86	0.54	1.51	43.00	1.43
4.9	2.92	0.59	1.62	40.00	1.43
5.0	3.01	0.65	1.80	41.00	1.41
5.1	3.15	0.74	1.99	41.00	1.41
5.2	3.24	0.83	2.22	40.00	1.31
5.3	3.54	1.12	2.72	42.00	1.19
5.4	3.69	1.38	3.20	43.00	1.32
5.5	3.93	1.78	4.05	44.00	1.26
5.6	4.72	4.49	7.82	43.00	1.91
5.7	5.23	7.17	11.09	45.00	1.29
5.8	5.64	8.99	15.30	44.00	1.11
5.9	5.81	9.55	16.19	43.00	1.60
6.0	6.26	14.67	22.64	43.00	1.65
6.1	6.95	17.22	28.50	49.00	1.10

Explanations:

basal end of the sample => section height: 0.0 mm

Sample 1			
**Section height**	**Cross-sectional area *A***	**Axial second moment of area *I***	**Polar second moment of area *J***
[mm]	[mm^2]	[mm^4]	[mm^4]
0.0	5.04	2.00	4.05
0.2	5.12	2.09	4.18
0.4	5.30	2.23	4.47
0.6	5.28	2.20	4.45
0.8	5.44	2.36	4.72
1.0	5.46	2.36	4.77
1.2	5.45	2.37	4.75
1.4	5.45	2.35	4.75
1.6	5.17	2.07	4.43
1.8	5.51	2.37	4.85
2.0	5.58	2.48	5.01
2.2	5.58	2.52	5.02
2.4	5.70	2.59	5.26
2.6	5.57	2.52	5.16
2.8	5.89	2.92	5.62
3.0	5.77	2.66	5.35
3.2	5.18	2.09	4.34
3.4	6.04	3.31	5.95
3.6	5.57	2.84	5.05
3.8	5.88	3.20	5.59
4.0	5.67	2.84	5.23
4.2	6.22	3.53	6.34
4.4	6.72	4.20	7.29
4.6	7.38	4.79	8.74
4.8	7.80	5.05	9.75
5.0	10.59	8.22	18.16
5.2	15.19	15.24	39.80
5.4	28.83	52.91	142.54

Sample 2			
**Section height**	**Cross-sectional area *A***	**Axial second moment of area *I***	**Polar second moment of area *J***
[mm]	[mm^2]	[mm^4]	[mm^4]
0.0	4.18	1.16	2.83
0.2	4.36	1.23	3.12
0.4	4.45	1.15	4.41
0.6	4.42	1.40	3.14
0.8	4.21	1.25	2.85
1.0	3.52	0.92	2.09
1.2	4.33	1.30	3.04
1.4	4.29	1.42	2.99
1.6	4.45	1.46	3.21

Sample 2 (continued)			
**Section height**	**Cross-sectional area *A***	**Axial second moment of area *I***	**Polar second moment of area *J***
[mm]	[mm^2]	[mm^4]	[mm^4]
1.8	4.39	1.35	3.14
2.0	4.35	1.32	3.05
2.2	4.34	1.37	3.02
2.4	4.36	1.34	3.05
2.6	4.45	1.47	3.19
2.8	4.37	1.37	3.08
3.0	4.39	1.43	3.10
3.2	4.39	1.39	3.10
3.4	4.29	1.38	2.94
3.6	4.24	1.35	2.88
3.8	4.26	1.34	2.92
4.0	4.49	1.46	3.23
4.2	4.52	1.50	3.27
4.4	4.65	1.52	3.47
4.6	5.05	1.66	4.18
4.8	5.29	1.78	4.60
5.0	6.22	2.24	6.54
5.2	6.64	2.55	7.68
5.4	10.26	6.44	21.99
5.6	17.12	18.08	55.62

Sample 3			
**Section height**	**Cross-sectional area *A***	**Axial second moment of area *I***	**Polar second moment of area *J***
[mm]	[mm^2]	[mm^4]	[mm^4]
0.0	5.67	2.25	5.18
0.2	5.77	2.33	5.36
0.4	6.25	2.51	6.43
0.6	6.02	2.54	5.84
0.8	5.81	2.37	5.44
1.0	5.57	2.10	5.03
1.2	5.57	2.18	5.00
1.4	6.00	2.43	5.84
1.6	5.86	2.38	5.53
1.8	6.23	2.63	6.30
2.0	5.97	2.36	5.80
2.2	6.13	2.39	6.16
2.4	6.37	2.68	6.59
2.6	6.85	3.19	7.60
2.8	6.57	2.89	6.99
3.0	6.77	3.10	7.40
3.2	6.88	3.20	7.65

Sample 3 (continued)			
Section height	Cross-sectional area *A*	Axial second moment of area *I*	Polar second moment of area *J*
[mm]	[mm^2]	[mm^4]	[mm^4]
3.4	6.86	3.23	7.60
3.6	6.93	3.41	7.73
3.8	7.03	3.55	7.92
4.0	6.93	3.61	7.67
4.2	7.35	4.18	8.63
4.4	7.61	4.53	9.25
4.6	7.83	5.38	10.08
4.8	7.69	5.14	9.48
5.0	7.35	5.09	8.73
5.2	7.40	4.63	8.87
5.4	8.06	6.04	10.50
5.6	8.45	6.49	11.50
5.8	12.70	15.35	27.06
6.0	21.75	42.01	76.70
6.2	42.00	141.75	308.43

Sample 4			
Section height	Cross-sectional area *A*	Axial second moment of area *I*	Polar second moment of area *J*
[mm]	[mm^2]	[mm^4]	[mm^4]
0.0	6.25	2.53	6.37
0.2	6.88	2.94	7.78
0.4	7.41	3.38	9.09
0.6	6.04	2.58	5.85
0.8	5.63	2.36	5.06
1.0	5.39	1.87	4.86
1.2	6.05	2.66	5.85
1.4	5.66	2.37	5.12
1.6	5.75	2.38	5.29
1.8	5.74	2.47	5.26
2.0	5.60	2.35	5.00
2.2	5.71	2.32	5.23
2.4	5.57	2.31	4.95
2.6	5.57	2.31	4.96
2.8	5.53	2.32	4.88
3.0	5.50	2.22	4.86
3.2	5.60	2.35	5.02
3.4	5.53	2.35	4.89
3.6	5.54	2.37	4.89
3.8	5.64	2.38	5.10
4.0	5.61	2.46	5.03
4.2	5.48	2.46	4.79

Sample 4 (continued)			
**Section height**	**Cross-sectional area *A***	**Axial second moment of area *I***	**Polar second moment of area *J***
[mm]	[mm^2]	[mm^4]	[mm^4]
4.4	5.97	2.77	5.69
4.6	5.73	2.59	5.26
4.8	5.45	2.25	4.78
5.0	5.81	2.75	5.40
5.2	6.06	3.01	5.87
5.4	5.91	2.88	5.59
5.6	5.81	2.71	5.40
5.8	6.16	2.93	6.05
6.0	5.97	2.83	5.70
6.2	6.22	2.75	6.27
6.4	6.67	2.95	7.30
6.6	12.49	13.09	30.38

Sample 5			
**Section height**	**Cross-sectional area *A***	**Axial second moment of area *I***	**Polar second moment of area *J***
[mm]	[mm^2]	[mm^4]	[mm^4]
0.0	5.05	1.80	4.12
0.2	5.28	1.89	4.51
0.4	5.13	1.74	4.28
0.6	5.12	1.80	4.23
0.8	5.07	1.82	4.14
1.0	5.25	1.88	4.46
1.2	5.32	1.90	4.58
1.4	5.33	1.97	4.58
1.6	5.30	1.88	4.55
1.8	5.27	1.98	4.48
2.0	4.56	1.25	3.53
2.2	5.29	1.97	4.50
2.4	5.43	2.06	4.84
2.6	5.67	2.14	5.23
2.8	5.38	2.10	4.64
3.0	5.67	2.27	5.20
3.2	6.21	2.96	6.45
3.4	4.38	0.99	3.94
3.6	5.58	2.19	5.05
3.8	6.58	2.87	7.39
4.0	6.36	2.79	6.63
4.2	5.62	2.42	5.21
4.4	4.57	0.96	4.12
4.6	5.45	1.71	5.20
4.8	5.94	2.41	5.76

Sample 5 (continued)			
Section height	Cross-sectional area *A*	Axial second moment of area *I*	Polar second moment of area *J*
[mm]	[mm^2]	[mm^4]	[mm^4]
5.0	5.64	2.10	5.45
5.2	6.48	2.82	6.88
5.4	12.92	31.17	43.76
5.6	24.55	37.21	189.23

Sample 6 (detailed serial section)					
Section height	Cross-sectional area *A*	Axial second moment of area *I*	Polar second moment of area *J*	Number of vascular bundles	Area fraction of the vascular bundles AF_v
[mm]	[mm^2]	[mm^4]	[mm^4]	[-]	[%]
0.0	7.84	6.94	10.45	5.00	7.20
0.1	10.69	13.38	19.69	6.00	6.07
0.3	11.66	16.55	23.77	6.00	6.87
0.4	11.33	15.10	22.09	8.00	5.15
0.5	12.12	17.56	25.65	9.00	4.98
0.6	7.59	6.49	9.76	8.00	5.62
0.7	11.47	14.98	22.50	8.00	4.63
0.8	11.26	14.74	21.78	8.00	5.27
0.9	7.47	6.38	9.49	7.00	5.54
1.0	10.36	11.94	18.06	8.00	5.85
1.1	7.22	5.69	8.75	6.00	6.17
1.2	10.05	11.39	17.13	7.00	5.54
1.3	10.57	11.96	18.62	7.00	5.43
1.4	10.64	12.43	19.12	7.00	5.63
1.5	9.91	10.73	16.48	7.00	5.42
1.6	9.63	9.74	15.43	6.00	5.90
1.7	10.71	12.32	19.26	6.00	6.20
1.8	8.32	7.14	11.42	6.00	6.35
1.9	9.72	9.59	15.53	7.00	5.72
2.0	9.68	9.49	15.38	7.00	6.36
2.1	10.32	10.91	17.54	7.00	6.92
2.2	8.30	6.87	11.25	8.00	6.08
2.3	8.54	6.63	11.80	9.00	7.66
2.4	8.73	7.55	12.45	7.00	7.49
2.5	6.66	4.23	7.22	8.00	6.61
2.6	9.42	8.12	14.31	10.00	5.56
2.7	10.86	10.87	19.10	9.00	5.44
2.8	11.25	11.38	20.44	10.00	4.76
2.9	11.80	11.96	22.44	10.00	4.31
3.0	12.00	12.47	23.06	10.00	4.35
3.1	12.13	12.44	23.62	10.00	3.48
3.2	13.34	15.07	28.51	10.00	4.03
3.3	13.16	14.47	27.75	9.00	3.24

Sample 6 (detailed serial section) (continued)					
**Section height**	**Cross-sectional area *A***	**Axial second moment of area *I***	**Polar second moment of area *J***	**Number of vascular bundles**	**Area fraction of the vascular bundles AF_v**
[mm]	[mm^2]	[mm^4]	[mm^4]	[-]	[%]
3.4	13.26	14.57	28.23	9.00	3.38
3.5	13.94	15.58	31.17	9.00	2.97
3.6	15.27	18.71	37.55	9.00	2.53
3.7	16.13	20.41	42.21	9.00	2.84
3.8	17.00	22.58	46.94	8.00	2.25
3.9	18.51	26.82	55.58	8.00	2.30
4.0	20.34	31.15	67.77	8.00	2.11
4.1	20.57	32.58	74.12	8.00	1.94
4.2	25.49	53.36	105.94	8.00	1.64
4.4	25.45	62.31	117.32	8.00	1.68
4.5	30.09	93.62	175.69	8.00	1.51
4.6	26.29	67.19	120.48	8.00	1.54
4.7	28.92	106.33	171.65	8.00	2.04

Explanations:

basal end of the sample => section height: 0.0 mm
